# Recent Development of Photodeformable Crystals: From Materials to Mechanisms

**DOI:** 10.34133/2021/9816535

**Published:** 2021-11-11

**Authors:** Cheng Huang, Rongjuan Huang, Simin Zhang, Haodong Sun, Hailan Wang, Beibei Du, Yuxin Xiao, Tao Yu, Wei Huang

**Affiliations:** ^1^Frontiers Science Center for Flexible Electronics (FSCFE), Shaanxi Institute of Flexible Electronics (SIFE) & Shaanxi Institute of Biomedical Materials and Engineering (SIBME), Northwestern Polytechnical University (NPU), 127 West Youyi Road, Xi'an 710072, China; ^2^Key Laboratory of Flexible Electronics (KLOFE) & Institute of Advanced Materials (IAM), Nanjing Tech University (NanjingTech), 30 South Puzhu Road, Nanjing 211816, China; ^3^Key Laboratory for Organic Electronics and Information Displays & Jiangsu Key Laboratory for Biosensors, Institute of Advanced Materials (IAM), Jiangsu National Synergetic Innovation Center for Advanced Materials (SICAM), Nanjing University of Posts and Telecommunications, 9 Wenyuan Road, Nanjing 210023, China

## Abstract

Photodeformable materials are a class of molecules that can convert photon energy into mechanical energy, which have attracted tremendous attention in the last few decades. Owing to their unique photoinduced deformable properties, including fast light-response and diverse mechanical behaviors, photodeformable materials have exhibited great potential in many practical applications such as actuators, photoswitches, artificial muscles, and bioimaging. In this review, we sort out the current state of photodeformable crystals and classify them into six categories by molecular structures: diarylethenes, azobenzenes, anthracenes, olefins, triarylethylenes, and other systems. Three distinct light-responsive mechanisms, photocyclization, *trans-cis* isomerization, and photodimerization, are revealed to play significant roles in the molecular photodeformation. Their corresponding photodeformable behaviors such as twisting, bending, hopping, bursting, and curling, as well as the potential applications, are also discussed. Furthermore, the challenges and prospective development directions of photodeformable crystals are highlighted.

## 1. Introduction

Stimuli-responsive materials have attracted enormous attention over the past few decades, due to their promising applications in sensing, bioimaging, data storage, deformation detectors, switches, security systems, etc. [1–4]. These smart materials are designed in a way that can change their physical or chemical properties when exposed to various external stimuli, such as external force [5], light irradiation [6], magnetism [7], heating [8], and pH [9]. Among the multitude of responses to stimuli, materials exhibiting mechanical effects are appealing and promising, in which the external energy can be transformed into the molecular macro- or micromechanical motions [10]. The covalent bond breaking, ring-opening/closing reactions, changes of molecular conformations, or packing modes may occur during the process. As one of the most available stimuli, light has predominant advantages such as easy access, nondirect contact, wide trigger range, and limited by-products. Depending on the targeted applications, light signal can also be controlled by multiple parameters like light wavelength, intensity, irradiative time, and polarization, which then induces a usable molecular property change [11, 12]. Therefore, increasing light-related technical achievements are emerging, and various optical responsive materials have been produced.

Among various optical responsive materials, photochromic materials have developed rapidly owing to their extensive application values in the fields of material science and information science, including optical information storage [13–15], optical recording [16], anticounterfeiting [17, 18], chemical sensors [19, 20], and biological imaging agents [21, 22]. Photochromism is a phenomenon that the color of a substance changes reversibly upon the light irradiation of a certain wavelength. Meanwhile, molecular structures and physical/chemical properties of the compounds will also be changed to a certain extent accordingly. The reversible photochromic phenomenon was firstly discovered in the 1960s [23]. Since then, scientists started profoundly investigating more photochromic compounds and tried to explore their application values. Organic photochromic materials can be mainly classified into three categories, diarylethenes [4, 24], azobenzenes [25], and spiropyrans [26] by molecular structures. Although photochromism is mainly induced by the change of molecular structures before and after irradiation, the mechanism is not the same. For example, the photochromic features of diarylethenes are derived from the formation of the pericyclic reactions. While the photochromic mechanism of azobenzenes is based on the *cis*-*trans* isomerization upon light irradiation, which shows great potential in the fields of drug storage and drug transportation [27]. Spiropyran compounds have the characteristics of rapid response, because the heterolysis of the C-O bond is at the level of picoseconds. In addition to the traditional application areas, some photochromic materials shine in fields such as superresolution fluorescence imaging [28], erasable writing [29], and visible light photoswitches [30]. The introduction of a new triarylethylene system with easy modification and fast response characteristics can realize the light-controlled morphology change of the materials. Furthermore, the wettability of the material surface can also be adjusted [31]. With photochromic systems being introduced increasingly, more fields will be explored in the future.

With the development of photochromic molecules, it was found that some photochromic materials not only exhibit a color change when exposed to light stimuli but also show mechanical effects in crystalline states [32–34]. This color-shape dual responsive phenomenon, namely, photodeformation, was firstly discovered by Lange et al. in rhodium(1) semiquinone complexes in 1992 [35], whose crystalline needles underwent a reversible bending about 45° when irradiated by near-infrared (NIR) light. This work has opened a door for a new branch of photoresponsive materials. However, there were lots of work only focusing on the investigation of materials that have the conversion from photon energy into electrical and electrochemical potential (photoelectric potential), but less on the conversion directly into mechanical energies. Generally, the occurrence of photoinduced mechanical phenomena, such as bending, twisting, and expansion, is mainly due to the existence of photochemically reactive groups which can undergo a diverse geometrical rearrangement under light irradiation. In many circumstances, this process is reversible by photoexcitation at different regions or heating at a certain temperature. In recent decades, many photodeformable systems have been successfully developed, including polymers [36–38] and molecular crystals [39]. For polymers, photodeformable phenomenon is quite common due to the advantages of high processability, flexibility, and corrosion resistance. Molecular crystals also exhibit many superior advantages such as simple synthesis, fast response, and short recovery time, which make them one of the most popular hotspots. However, compared to polymers, only a few photodeformable crystals were reported, due to the inadequate understanding of the inherent mechanism and molecular design strategy.

Upon light exposure, a large diversity of mechanical motions can be observed, mostly deriving from the change of molecular structures or packing modes. Therefore, different photosensitive chromophores may have different mechanisms for various photoresponsive behaviors [40]. According to literatures, three types of mechanisms that may induce photodeformation were reported: photocyclization, *trans*-*cis* photoisomerization, and photodimerization. Photocyclization is an intramolecular photochemical process, with the transition from open-ring to closed-ring isomer by forming a new single bond when exposed to light irradiation. This process occurs accompanied by the change of molecular structures; the anisotropic change in the stacking pattern of crystal further leads to the mechanical deformation. *Trans*-*cis* photoisomerization refers to a reversible *trans*-*cis* isomerization process induced by light excitation, which is often accompanied by perturbation of the crystal arrangement leading to macroscopic deformation. For photodimerization, it is a photochemical dimerization process, including [2 + 2] and [4 + 4] cycloaddition reactions. A significant change in the size and arrangement of original molecular crystals will occur during the photodimerization process, which may lead to the micro- or macromechanical changes. Overall, photodeformable process is closely related to molecular structures and crystal arrangements.

All the excellent properties endow photodeformable materials with tremendous attentions in both academia and industry, representing their future trend in many applications [41–43] including artificial muscles [44], soft robots [45], biomedical devices [46], and photoswitches [42]. For example, the diverse photoinduced motions of photodeformable materials can mimic the movement of creatures, such as crawling, creeping, or jumping. Some can behave like human arms showing grasping or holding behaviors [44]. Their remarkably brilliant properties also give them great potential in biomedical field [41], such as delivering targeted drug and stimulating tissue regeneration. In addition, microrobots or microsensors have been achieved by employing micro- and nanosized photodeformable crystals [47]. These excellent properties and application prospects prompt us to assess and review the current status of photodeformable materials.

**Figure 1:**
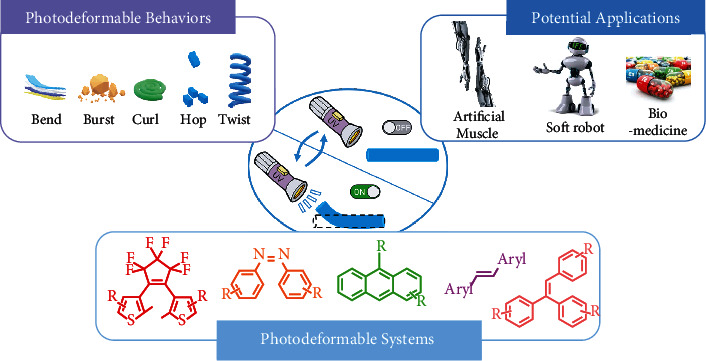
Representative examples of photodeformable systems, behaviors, and potential applications.

In this review, different types of photoinduced deformation systems are categorized according to their molecular structures. A variety of molecular examples on the latest progress as well as photodeformable behaviors and their potential applications are presented showing a comprehensive review. The commonly used building blocks for photodeformable materials include diarylethenes, azobenzenes, anthracenes, olefins, triarylethylenes, and some other systems, as shown in Figure 1. Diarylethenes and triarylethylenes tend to undergo a reversible photocyclization process upon light irradiation. While azobenzenes are typical compounds for photoinduced *trans*-*cis* isomerization. For anthracenes and olefins, another common photodeformable mechanism of photodimerization play an important role. By summarizing these systems, we aim to present the structure-property relationship of these photomechanical materials in details and try to figure out the following questions: (i) is it possible to control the light intensity and exposure time to manipulate the mechanical direction and magnitude? (ii) how to manipulate the molecular level motions to achieve the large-scale mechanical change? (iii) what new applications can be derived from the materials? Finally, the challenges and possible development directions in the future are also discussed.

## 2. Diarylethene-Based Photodeformable Materials

**Scheme 1:**
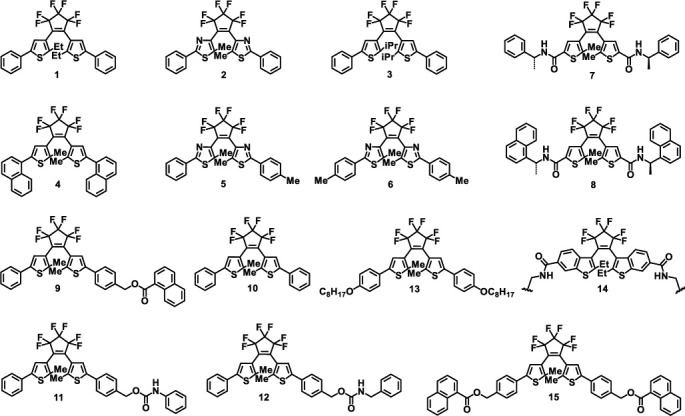
Molecular structures of diarylethene derivatives discussed in this section.

Diarylethene-based photochromic and photodeformable materials are a class of compounds containing five-membered heterocyclic rings, which have great thermal stability and high sensitivity. They have been widely reported as potential and indispensable candidates in the fields of optical memory [48] and molecular machines [49]. Under alternating UV and visible light irradiation, diarylethene derivatives tend to undergo reversible ring-closure and ring-open reactions. Because of the dense accumulation of molecules in the crystal lattice, crystals are likely to deform macroscopically accompanying their photochromic processes. The chemical structures of diarylethene molecules discussed in this section are shown in Scheme 1.

**Figure 2:**
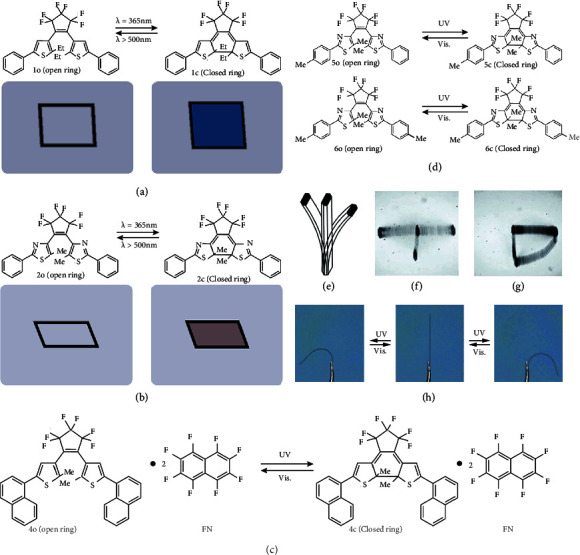
(a) Photochromic reaction and deformation of 1. (b) Photochromic reaction and deformation of 2. (c) Photochromic reaction of 4o·FN cocrystal. (d) Photochromic reactions of 5o and 6o. (e) Schematic illustration of bending of the rodlike crystal composed of 5o and 6o (5 : 6 = 63/37). (f) The bending of crystal under the different directions (left, right, and lower) of UV light. (g) The rotation of the edge of crystal upon controlled-intensity UV and Vis light irradiation. (h) Reversible deformation of crystal upon alternate irradiation of UV and visible light (adapted and copyright permission [53] (e–h), Wiley-VCH Verlag GmbH & Co. KGaA).

In 2007, two photodeformable single crystals based on diarylethenes were reported by Kobatake and coworkers [50]. As shown in Figure 2(a), 1,2-bis(2-ethyl-5-phenyl-3-thienyl)perfluorocyclopentene (1) exhibited not only a color change from colorless to blue but also a shape change from square to diamond under UV irradiation. By replacing thiophene to thiazole substituents, single-crystal 1,2-bis(5-methyl-2-phenyl-4-thiazolyl)perfluorocyclopentene (2) exhibited similar properties. A reversible color change from colorless to purple and reversible shrinkage and expansion of the rectangular plate-shaped single crystal of up to 7% were observed under UV and visible irradiation (Figure 2(b)). The deformation process took only 25 microseconds. In addition to the plate-shaped crystals, rod-shaped crystals that can bend under UV irradiation were also prepared. The bending tended to occur towards the direction of the incident UV light, which was attributed to the gradient of the photoisomerization degree. In other words, the accumulation of transformation in each diarylethene molecule leads to the macroscopic deformation of the crystals [34]. Based on previous work, they further reported another isopropyl-substituted crystal, 1,2-bis(2-isopropyl-5-phenyl-3-thienyl)perfluorocyclopentene (3) [51]. All these three crystals with similar crystal structures and molecular stacking forms exhibited similar photoinduced deformation processes.

In 2010, the same group designed a two-component photodeformable cocrystal, consisting of a photochromic diarylethene derivative (4) and perfluoronaphthalene (FN) [52]. The photochromic reaction of 4o·FN cocrystal was depicted in Figure 2(c), showing a reversible photocyclization reaction. The cocrystal with a length of 1-5 mm showed a reversible bending motion under the alternating UV and visible irradiation, which can be repeated more than 250 times. Single-crystal analyses indicated that the bending deformation of 4o·FN cocrystal was due to the anisotropic expansion of diarylethenes during the photocyclization process. Furthermore, molecular crystal cantilevers consisting of 4o·FN showed amazing performances in practice, which can lift a metal ball 200-600 times heavier than itself. The successful conversion of light into mechanical work suggested a promising application in micro- and nanomechanics, such as direct biological cells and light-driven valve manipulation in microreactors.

In order to improve the fatigue resistance, the idea of preparing the mixture was proposed by Terao et al. in 2011 [53]. The photoinduced reactions and schematic illustration of rod-shaped hybrid crystals 5o and 6o are shown in Figures 2(d) and 2(e), which showed reversible and rapid bending even with high fatigue resistance upon alternating UV and visible irradiation. Regardless of the direction of incident light, the rodlike crystal always bent to the UV light source (Figure 2(f)). By controlling the light intensity, the edge of the rod crystal rotated (Figure 2(g)). The photoinduced bending can also occur in water and a low temperature. Moreover, rodlike crystal can bend and strike the gear under UV light, leading to the rotation of gears. While upon visible light irradiation, the crystal changed back to its initially straight shape. Then, the UV rays bent the crystal, and the gears rotated again (Figure 2(h)). The crystal had the same mechanical properties as the piezoelectric crystal, which showed great potential in diverse micro- and nanomechanical applications.

**Figure 3:**
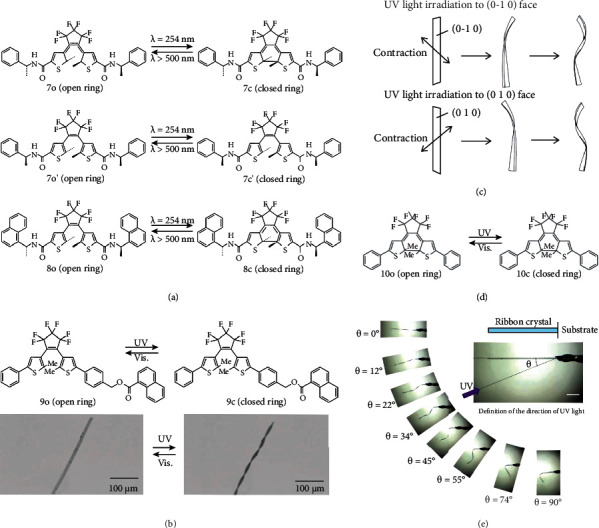
(a) Photochromic reaction of 7o, 7o′, and 8o. (b) Photochromic reaction and reversible phototwisting of 9o upon UV and Vis light irradiation. (c) Relationship between the direction of twisting and the face irradiated with UV light (adapted and copyright permission [54] (b, c), Wiley-VCH Verlag GmbH & Co. KGaA). (d) Photochromic reaction of 10o. (e) Different twisting motions caused by controlling the angle of incident light (adapted and copyright permission [55], American Chemical Society).

Some researchers have been devoted to investigating the factors that lead to different photoinduced deformation behaviors of the same crystal. Back to 2008, Uchida and his colleagues found that reversible photoinduced bending of crystals can be achieved in both convex and concave forms [56]. They reported the photochromic reactions of three crystals, 7o, 7o′, and 8o (Figure 3(a)). The lamellar crystal of 7o can be rolled up upon 254 nm UV irradiation, and the bending can occur within 3 s. It can recover when the UV light was moved away. It revealed that the response time to the light irradiation was strongly dependent on the light intensity. Similarly, thin crystal 8o also showed rollup under UV irradiation, and the bending angle was related to the thickness of the crystal. The bending behavior was attributed to the change in the stacking pattern of the crystal surface. Therefore, it is of great significance to dig into the change of surface morphology under the alternate UV and visible light irradiation to better understand the mechanisms of photodeformation.

In 2013, a diarylethene crystal (9o) showing photoinduced twisting behaviors was reported by Kitagawa and coworkers (Figure 3(b)) [54]. The twisting can occur in two directions, the left-hand helix and the right-hand helix, depending on the irradiated crystal surfaces of (010) or (0-10) faces. While 9o contracted when the whole crystal was exposed to the UV irradiation from both surfaces. In 2018, similar twisting behaviors have also been reported in a ribbon-like crystal of diarylethene derivative (10o) by Kitagawa et al. (Figure 3(d)) [55]. The photomechanical twisting can be changed by tunning the irradiation directions. As shown in Figure 3(e), the banded crystal could be further twisted into a helicoid shape by irradiation from the top (incident light angle *θ* of 0°). By gradually increasing irradiation angles, crystal 10o could progressively change into a cylindrical helix shape. When it was increased close to 90°, 10o was bent instead of twisting. The experimental results indicated that the irradiation direction can manipulate the stress tensor of crystal surface. Although the mechanism of this phenomenon still needs to be studied, the incident angle could be used to control the photoinduced deformation, which lays a solid foundation for further study of photodeformable devices.

**Figure 4:**
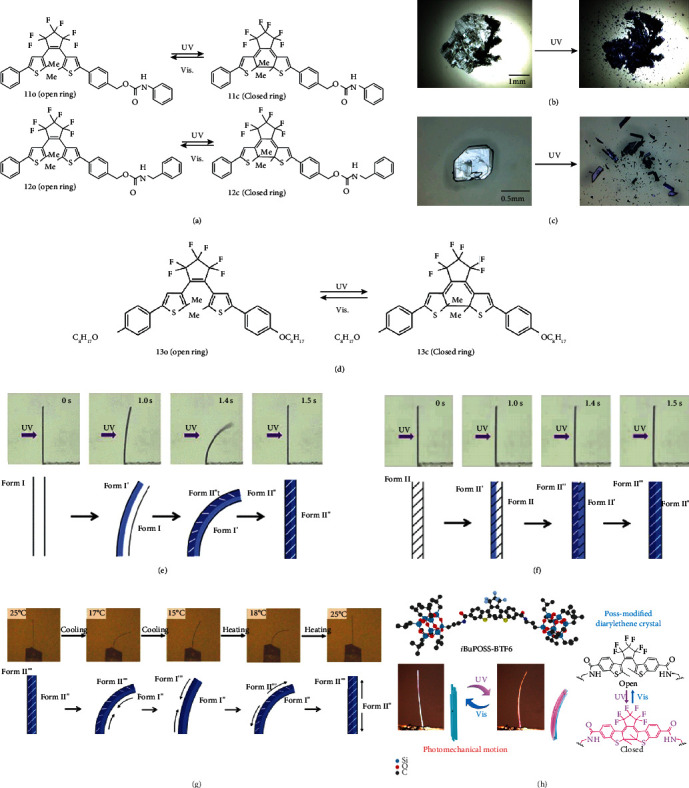
(a) Photochromic reactions of 11 and 12. (b) Photographs of crystal 11 before and after UV irradiation. (c) Photographs of crystal 12 before and after UV irradiation (adapted and copyright permission [57] (b, c), American Chemical Society). (d) Photochromic reaction of crystal 13o. (e) Photoinduced behavior of crystal 13o upon UV irradiation at 25°C. (f) Photoinduced behavior of crystal 13o upon UV irradiation at 66°C. (g) Bending behaviors of the photoirradiated crystal 13o by controlling temperature (adapted and copyright permission [58] (e–g), American Chemical Society). (h) The structure of POSS-modified diarylethene crystal (14) and its photomechanical motion (adapted and copyright permission [59], American Chemical Society).

Various photodeformable behaviors, such as bending, twisting, curling, shrinkage, and expansion, have been reported in a series of diarylethylene crystals. In 2016, a photoinduced rapid explosive process was reported in the photochromic process of diarylethylene crystals 11o and 12o (Figure 4(a)) [57]. Figures 4(b) and 4(c) show the photographs of crystals 11o and 12o. Upon UV irradiation, the color of both crystals changed from colorless to blue. In addition, they rapidly burst into pieces unexpectedly while retaining their original excellent photochromic properties. It was proposed that the remaining fragments after exploration were mainly due to intermolecular hydrogen bonds between the urethane units.

In 2017, Kitagawa et al. found that both photochromic reaction and reversible thermodynamic phase transition can trigger photoinduced bending behaviors with good repeatability in a diarylethene derivative crystal 13o [58]. The photochromic reaction of crystal 13o is shown in Figure 4(d). At room temperature, crystal 13o firstly bent slowly away from the incident UV light, which suddenly bent sharply under continuous UV irradiation. It was due to the thermodynamic phase transition occurring in the photoreacted crystal surface layer (Figure 4(e)). Upon further UV irradiation, the crystal suddenly returned to its original linear shape. However, when the temperature increased to 66°C, crystal 13o barely bent (Figure 4(f)). It was proposed that the unique mechanical behavior of 13o was induced not only by the photochromic reaction but also by the reversible single-crystal-to-single-crystal phase transition. Furthermore, the crystal after continuous UV irradiation at room temperature, namely, photoirradiated crystal, would bend when it cooled down. This process was reversible after heating as depicted in Figure 4(g). The experimental results showed that the thermal effect is also one of the factors of the abnormal bending behavior. This work provides a new direction for subsequent research on photodeformable crystals.

Except for the aforementioned photodeformable materials based on pure molecular crystals, the combination of organic crystals and inorganic components is also a promising way to design photodeformable materials. In 2019, Kajiya and coworkers explored the properties of diarylethene siloxane hybrid, inspired by previously reported azobenzene siloxane hybrid [59]. To combine the excellent properties of silsesquioxane and diarylethene, two polyhedral oligomeric silsesquioxane (POSS) cages were used to modify diarylethene derivatives containing benzothiophene ring, and a new type of organic-inorganic hybrid photodeformable material was prepared. By introducing POSS, the conversion of closed-loop diarylethene isomer in solution was increased upon UV irradiation. Meanwhile, the long needle-like hydrogenated molecular crystals can be obtained, which was attributed to the amide bond connection between diarylethene and POSS units. These crystals have better thermal stability, reversible photochromism, and photomechanical response. From Figure 4(h), it can be observed that the POSS-modified diarylethene crystal performed both reversible photochromism and photoinduced bending upon UV and visible light irradiation. The high fatigue resistance of diarylethenes combined with the enhanced thermal stability caused by the siloxane groups makes these crystals a possibility for actuators and other smart devices.

**Figure 5:**
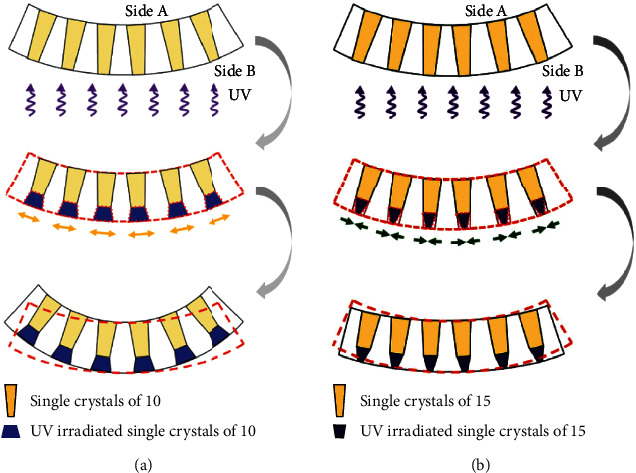
Photodeformation of single crystals (a) 10 and (b) 15 (adapted and copyright permission [60] (a, b), Wiley-VCH Verlag GmbH & Co. KGaA).

Later on, Dong and his colleagues tried a few methods to characterize composites composed of inorganic and organic components to explore the influence of nanostructures on the photomechanical properties [60]. They developed a new organic-inorganic photomechanical actuator consisting of diarylethene nanowire crystals and anodic aluminum oxide (AAO) porous templates. The nanocrystals grew in the internal channels of AAO porous templates.  Figure 5 presents two different photodeformable behaviors of crystals 10 and 15 associated with photoisomerization. Upon UV irradiation, crystal 10 exhibited a molecular expansion, giving an increase of the curvature. In contrast, crystal 15 showed a contraction and a curvature decrease. By combining inorganic framework and active organic component in the nanostructures, the photoinduced properties of these organic-inorganic hybrid composites have been ameliorated manifestly, which also have considerable room for improvement.

## 3. Azobenzene-Based Photodeformation Materials

**Scheme 2:**
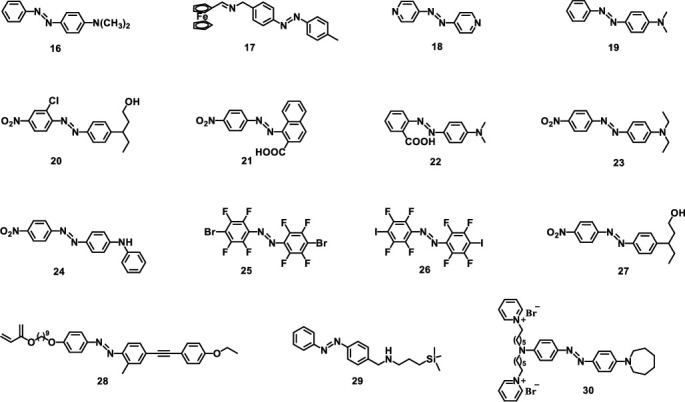
Molecular structures of azobenzene derivatives discussed in this section.

Azobenzene is a diazene (-HN=NH-) derivative in which two hydrogen atoms are replaced by phenyl groups. It is a typical photochromic and photodeformable system owing to the reversible *trans-cis* isomerization processes driven by light irradiation. This isomerization might lead to the deformation of the crystals in shapes and sizes [61]. In addition, the geometric changes of these azobenzene derivatives may lead to crystal motions due to the accumulated strain in the crystalline states. The sensitive photodeformable responses of these crystals upon UV, visible, or near-infrared light irradiation give them broad application prospects particularly in biomedical area. The chemical structures of azobenzene-based molecules discussed in this section are listed in Scheme 2.

**Figure 6:**
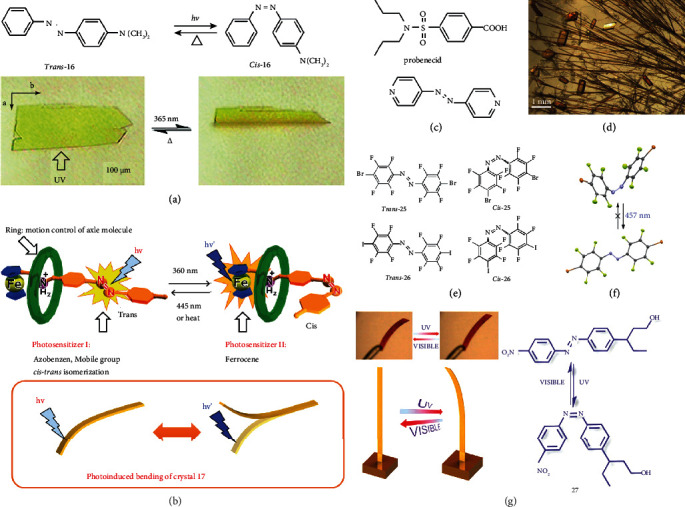
(a) Chemical structure of trans-16/cis-16 and its photomechanical motion upon UV irradiation (adapted and copyright permission [62], American Chemical Society). (b) The *trans*-*cis* isomerization of azobenzene-containing pseudorotaxanes and photoinduced bending of 17 (adapted and copyright permission [63], American Chemical Society). (c) Molecular structures of probenecid and 18. (d) Microscopic photographs of the cocrystal, acicular and blocky crystals (adapted and copyright permission [64], Wiley-VCH Verlag GmbH & Co. KGaA). (e) Chemical structure of trans-25/cis-25 and trans-26/cis-26. (f) Single-crystal structures of cis → trans isomerization of 25 (adapted and copyright permission [66], American Chemical Society). (g) Chemical structure of crystal 27 and its photomechanical motion (adapted and copyright permission [67], American Chemical Society).

The photoinduced bending behaviors of azobenzene crystals were firstly reported in platelike microcrystals of *trans*-4-(dimethylamino) azobenzene (*trans*-16) by Koshima and coworkers in 2008 [62]. Upon UV irradiation, *trans*-16 showed a reversible bending manner (Figure 6(a)). It can bend quickly to the opposite direction of the incident light and the maximum deflection was achieved after 0.5 s. After stopping the irradiation, it can return to its initial shape within 30 s. In addition, the roughness of (001) top surface of *trans*-16 also changed after UV irradiation as shown by atomic force microscopy (AFM), which was proposed due to the photoisomerization to *cis*-16 on (001) surface. In *cis*-isomer, the repulsion between the two phenyl planes induced an increased torsional conformation. Other surfaces did not undergo photoisomerization without light irradiation. These changes resulted in the bending of the microcrystal. In 2018, Cheng et al. reported another photodeformable azobenzene crystal, pseudorotaxane (17), which showed fast and highly reversible bending properties [63]. The dynamic light response of crystal 17 containing azobenzene and ferrocene groups in the axial conformer was studied, as shown in Figure 6(b). The results indicated that the pseudorotaxane containing methylazobenzene-based host-guest supramolecular compound can undergo a reverse bending of 20-30° by alternating UV and visible light irradiation. It showed a fast response of ca. 0.3 s, which is closely due to the strong intermolecular *π*-*π* interactions of the azophenyl group. In general, pseudorotaxane molecules can assist the bending of crystals, which provide the adjustability of molecular structure changes, and allow some flexibility in the mechanical movement of crystals. These dynamic crystals showed great potential in the applications of molecular actuators or photoswitches.

In addition to single crystals, photodeformation was also explored in intelligent eutectic. In 2018, Gupta and coworkers [64] developed a plastically bendable probenecid molecular crystal cocrystallized with 4,4′-azopyridine (18) in a ratio of 2 : 1. This cocrystal exhibited bending, twisting, and elastic deformation without molecular fracture under various external stimuli, such as UV light, heat, and mechanical pressure. Optical microscopic images showed that the cocrystal had two polymorphs, acicular crystals and blocky crystals (Figure 6(d)). Both of them can bend reversibly upon UV irradiation and show elastically deformed behavior under mechanical force.

In photodeformable crystals, the photomechanical motions are mostly driven by UV light while few by visible light. In 2013, Bushuyev et al. reported several visible-light-driven photodeformable crystals, pseudostilbenes (19-24), by attaching strong “pull” electron-withdrawing and “push” electron-donating groups to azobenzene moieties [65]. It indicated that the push-pull electronic ability in the crystals enhanced their photodeformable properties. In addition, the lower bulk density and thinner thickness could also significantly promote their photoinduced bending. Later on, the same group further developed two more visible-light-driven photodeformable crystals, perhalogenated *cis*-azobenzene (*cis*-25 and *cis*-26) (Figure 6(e)) [66]. Both crystals can undergo a crystal-to-crystal *trans-cis* isomerization for its unusually long thermal half-lives. Different from the previously studied *trans*-azobenzene system, which could not achieve permanent bending due to the rapid equilibrium formed after stoppage of irradiation, *cis*-25 and *cis*-26 could transform into a polycrystalline aggregate of their *trans*-isomer upon visible light irradiation. This *cis-trans* transformation involved significant, controllable, and thermally irreversible changes of the crystal shapes. These two crystals were the first cases showing permanent photodeformation in azobenzene systems. It was proposed that the crystal thickness or efficient molecular packing modes of *trans*-azobenzene crystals are the critical factors for permanent bending. This work opens up a new strategy to design permanently photodeformable azobenzene solids.

In the beginning, the qualitative bilayer model was used to clarify the mechanisms of the photodeformable behaviors in azobenzenes. However, this model was unable to analyze both internal and external factors on the photodeformable properties. In 2014, Nath and coworkers proposed a new dynamic model, by using a simple mathematical description to depict the quasistatic and time-varying photobending of the macroscopic single crystals [67]. In this work, the photoresponsive azobenzene-dye Disperse Red 1 (27) was studied by the new model, and the monotonic bending and recovery of its elongated crystal were successfully analyzed, as shown in Figure 6(g). The model can be used to explain not only the dynamics of bending and straightening but also the dependence of deflection angle on excitation dose. It was clearly proved that the dynamics of photoinduced bending depended on both internal factors (crystal size) and external factors (irradiation excitation, intensity, and direction).

**Figure 7:**
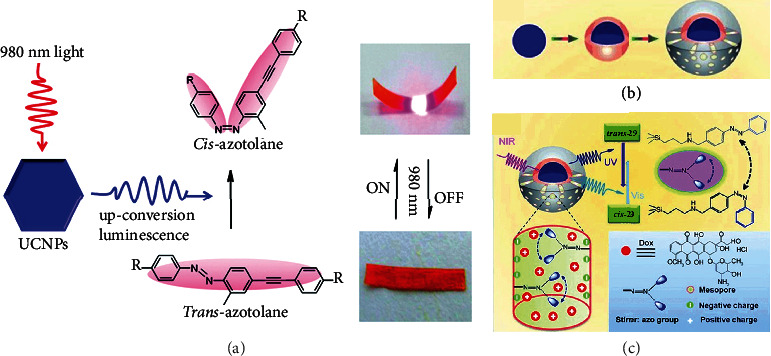
(a) Photographs of bending of composite film under 980 nm light (adapted and copyright permission [68], American Chemical Society). (b) Synthesis of upconverting nanoparticles with integrated azobenzene-modified mesoporous silica. (c) The process diagram of photomechanical movement of upconverting nanoparticles with azo-modified mesoporous silica (adapted and copyright permission [69] (b, c), Wiley-VCH Verlag GmbH & Co. KGaA).

Except for pure azobenzene crystals, azobenzene derivatives are often combined with gels and liquid crystals forming photodeformable systems, which exhibit promising potential in biomedical field. However, photodeformable materials that need to be driven by UV light are not suitable for application in cells or organisms, mainly due to the destructiveness of high excitation energy. Therefore, it is of great significance to develop photodeformable materials that can be driven by visible light or NIR light. In 2011, Wu et al. reported a composite film by incorporating upconversion nanophosphors (UCNPs) into azobenzene (28)-containing polymer films (Figure 7(a)). A fast bending behavior upon continuous-wave NIR light irradiation at 980 nm was achieved [68]. It was mainly due to the synergistic effect of *cis-trans* photoisomerization of 28 and the alignment change of the mesogens. Another example was reported by Liu and coworkers in 2013, by combining an upconverting nanoparticles with integrated azobenzene (29)-modified mesoporous silica [69]. Figure 7(b) shows the synthesis process, with the anticancer drug doxorubicin packing into the mesopores. After absorbing NIR light (980 nm), nanoparticles emitted photons in the UV/visible region. These photons were absorbed promptly by azobenzene molecules and then transferred to their continuous rotation-inversion motions. The photoinduced motions of azobenzene molecules facilitated the release of anticancer drug, and nanoparticles were in charge of delivering the anticancer drug as depicted in Figure 7(c). The drug release can be well controlled by the irradiation intensity or time of NIR light. Recently, Paternò et al. found an amphiphilic azobenzene derivative (30) with noncovalent affinity for cell membranes [70]. Unlike some other photochromic molecules, molecule 30 was dephotoisomerizable in water. It also showed fast response in cells, enabling it to regulate the potential of the cell membrane fast and reversibly. This work showed great potential for the development of photopharmacology and biophotonics.

## 4. Anthracene-Based Photodeformable Materials

**Scheme 3:**
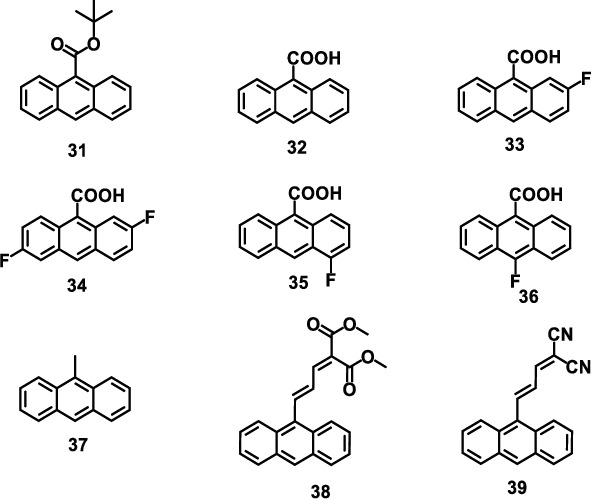
Molecular structures of anthracene derivatives discussed in this section.

In addition to diarylethenes and azobenzenes, some crystals of anthracene derivatives also exhibit photodeformable behaviors. Reversible photocycloaddition reactions are crucial for anthracene crystals to perform various photomechanical motions, including bending, twisting, curling, and jumping. In anthracene molecules, the double bonds at 9, 10-positions are photoactive for the [4 + 4] photocycloaddition reactions, provided they could meet the conditions of parallel molecular packing and proper distance between two anthracene planes satisfying the Schmidt rules. The chemical structures of anthracene derivatives discussed in this section are shown in Scheme 3.

In 2006, Al-Kaysi and coworkers found the photochemically driven shape changes in crystalline nanorods 9-tert-butylanthroate (31) [71]. Upon 365 nm irradiation, nanorods 31 emitted in a wide yellow-green region, which bent rapidly and recovered subsequently when light intensity was increased to 70 mW/cm^2^. In addition, the millimeter and micron-scale crystals showed anisotropic expansion which then decomposed to smaller irregular crystals. The results indicated that the crystalline shape changes were mainly attributed to the crystal-to-crystal photodimerization. The single-crystal analyses indicated that the volume per anthracene moiety showed an increase of 9.7%, changing from 371 A^3^ in the monomer crystal to 407 A^3^ in the dimer crystal. In addition, they tried to test the reversibility of dimer with short wave UV light. However, the crystal dimensions did not shrink back to their initial state. Based on the previous work, they used a new model, by combining ensemble oriented-crystal solid-state NMR, X-ray diffraction, and first principle computational modeling, to figure out the atomic resolution and molecular mechanism of nanorods 31 with photoinduced expansion [72]. It revealed that that the photoexpansion of nanorods resulted from an anisotropic rearrangement of molecular content, not the variation of the unit cell volume.

Subsequently, Al-Kaysi and Bardeen demonstrated that the use of an intermolecular photochemical reaction can achieve large physical displacements in a different type of structure, organic molecular crystal nanorods [73]. The molecular crystal (32) nanorods can undergo a reversible photoinduced cycle between clear shapes after being excited. This work also confirmed that the nanoscale molecular crystal structure can reduce the damage and fragmentation caused by strain, which is usually related to the photochemical changes in larger (micron to millimeter scale) crystals. However, smaller crystals need to be synthesized in further study. Afterwards, they engaged in the exploration of how to control the size, rate, and direction of the photoinduced deformation in nanorods by using laser excitation conditions in 2009 [74]. The reported molecular crystal nanorods are composed of 9-anthracene carboxylic acid (32), whose bending mechanism was figured out to be the lattice mismatch between the monomer and dimer when exposed to light. The highly focused NIR femtosecond laser pulses can trigger a two-photon excitation in the micron-scale region, which would induce the transient bending at different positions along the length of a 200-nanometer single nanorod. In addition, the position of bending was controlled by the laser spot position, and the size and direction were controlled by the laser intensity and/or the exposure time. It indicated that the photodeformable effects in molecular crystal nanorods can be manipulated in a repeatable and precise way, which brings them a broad application prospect in the future. Overall, the twisting behavior of crystal microstrip 32 upon UV irradiation was induced by [4 + 4] photodimerization reactions. However, some basic questions, for instance, whether heterogeneous domains of reactant and product can form within the crystal, still remain unclear.

**Figure 8:**
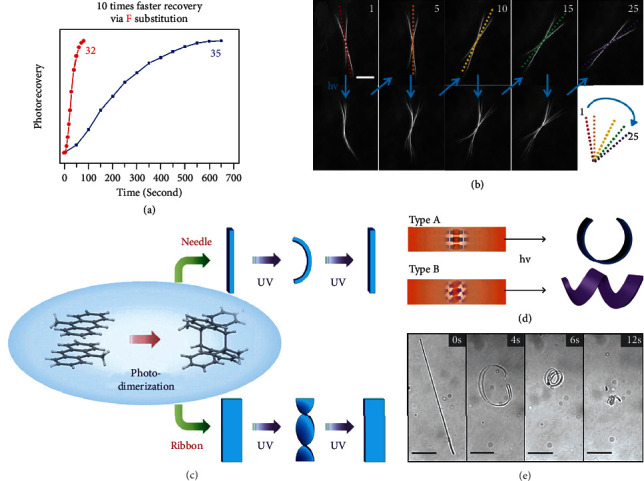
(a) The contradistinction of photorecovery time between 32 and 35 (adapted and copyright permission [75], American Chemical Society). (b) The clockwise rotation of the X-shaped crystal 35. The cycle was repeated for 25 times (adapted and copyright permission [76], Wiley-VCH Verlag GmbH & Co. KGaA). (c) Two kinds of photomechanical behaviors of 37 (adapted and copyright permission [77], American Chemical Society). (d) Two types of photomechanical motion generated by crystal 37 microplates (adapted and copyright permission [78], Wiley-VCH Verlag GmbH & Co. KGaA). (e) Snapshots of curling of an (E)-38 nanowire (adapted and copyright permission [79], Wiley-VCH Verlag GmbH & Co. KGaA).

Although it has been well known that crystal 32 shows a reversibly photodeformable response, it has several drawbacks: (i) it is unstable under irradiation, and only 10 cycles can be maintained before losing a significant amount of response; (ii) it has a slow dimer dissociation time (∼400 s), lowing the duty cycle of an actuator; (iii) it is relatively brittle and tends to crack or shatter under light. To circumvent these problems, Zhu et al. reported a few fluoro-substituted derivatives based on 32 in 2014, as fluorine has a close size to hydrogen and can minimize the size-induced disruptions [75]. These crystals were 2-fluoro-9-anthracene carboxylic acid (33), 2,6-difluoro-9-anthracene carboxylic acid (34), 4-fluoro-9-anthracene carboxylic acid (35), and 10-fluoro-9-anthracene carboxylic acid (36). These derivatives have similar molecular electronic structures and crystal stacking modes, while different photoinduced mechanical properties. Particularly, 35 showed a better recovery time and circulation ability than 32 (Figure 8(a)). It is supposed that the improvement of photomechanical properties was attributed to the change of intermolecular *π*-*π* interaction or hydrogen bond.

With the in-depth study of photomechanical crystals, how to effectively utilize the motion induced by light has become one of the main research directions as well as one of the challenges in this field. Driven by pH stimulus, the slow reprecipitation of 35 in aqueous solution brought about the growth of branched microcrystals [76]. When these microcrystals were irradiated with light with a wavelength of 405 nm, the [4 + 4] photodimerization between molecules caused the twisting and bending of a single branch, thus driving the rotation of the whole crystal. This rotation can be repeated, resulting in a ratchet-like rotational motion. Figure 8(b) shows a sequence of images that rotated clockwise after each irradiation period. The angle and direction of rotation varied depending on the shape of the crystal, such as the number, length, and direction of branches. Further study indicated that the chiral shape of these crystals was the main reason for the ratchet-like rotation.

To explore the origin of the directional motions, Kim and his team engaged on the investigation of the solid-state [4 + 4] photodimerization reaction in crystal 9-methylanthracene (37), trying to figure out how the crystal morphology and photodimerization dynamics affect its deformation in 2014 [77]. By changing the crystallization conditions, two kinds of crystals, microneedles and microribbons, were obtained, which have the same crystal orientation. However, they showed different photoinduced deformation behaviors under UV radiation, i.e., microneedle crystal bent while microribbon crystal twisted, as shown in Figure 8(c). The deformation reached maximum at the midpoint, instead of the end, of the reaction. It was demonstrated that the morphology and reaction dynamics of the crystals affect the photoinduced deformation ability of the crystal, which need to be taken into consideration in the design of molecular crystal photomechanical elements.

Based on previous work, Tong et al. employed a new surfactant-assisted seeded-growth method to grow single-crystal platelets consisting of 37 in 2018 [78]. It showed two different internal molecular orientations, giving two optical-mechanical responses. Stable type A showed a photoinduced rolling up and unfolding, while only twisting behavior was observed in the less stable one (Figure 8(d)). The rolled-up cylinder can capture superparamagnetic nanoparticles, leading to its movement in a magnetic field. This special property provides a possibility to control the translational motion of crystals by light.

Besides crystals 32 and 37, some other anthracene derivatives with (E)- and (Z)-isomers were also reported. Anthracene derivative 38 is very stable at darkroom temperature and can be easily crystallized in different organic solvents [79]. There is no strong intermolecular interaction between the (E)- and (Z)-38 isomers. When the single-crystal nanowires grew, the photoisomerization reaction led to a great change in the shape. Figure 8(e) shows the SEM images of diameter nanowires. It can be observed that the nanowire curled immediately and then lasted for a few seconds, until it collapsed into a tightly coiled ball. Unlike the previous mechanism of photoinduced twisting, the curling phenomenon in 38 required both reactants and product species, which was consistent with the crystalline heterogeneity mechanism. The curling was driven by the generation of amorphous product phase instead of a crystal-to-crystal phase transition. In addition, another new anthracene derivative (39) with photodeformable responses was reported by Zhu et al., which exhibited a strong charge-transfer transition [80]. The E → Z isomerization reaction of the molecule occurred rapidly upon visible irradiation and then recovered under UV light. The reversible transition can induce reversible shape change, such as bending of microribbon crystals and nanowire crystals.

## 5. Olefin-Based Photodeformable Materials

**Scheme 4:**
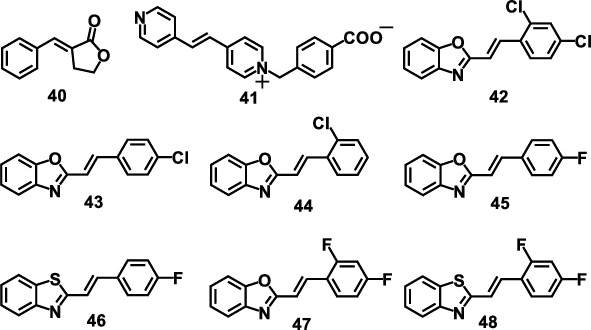
Molecular structures of olefins discussed in this section.

Apart from [4 + 4] reactions, [2 + 2] photocycloaddition reactions of olefins can also lead to the photoinduced motions. The photoreaction mechanism of olefins resembles that of anthracenes. The molecular structures of olefins discussed in this section are shown in Scheme 4.

**Figure 9:**
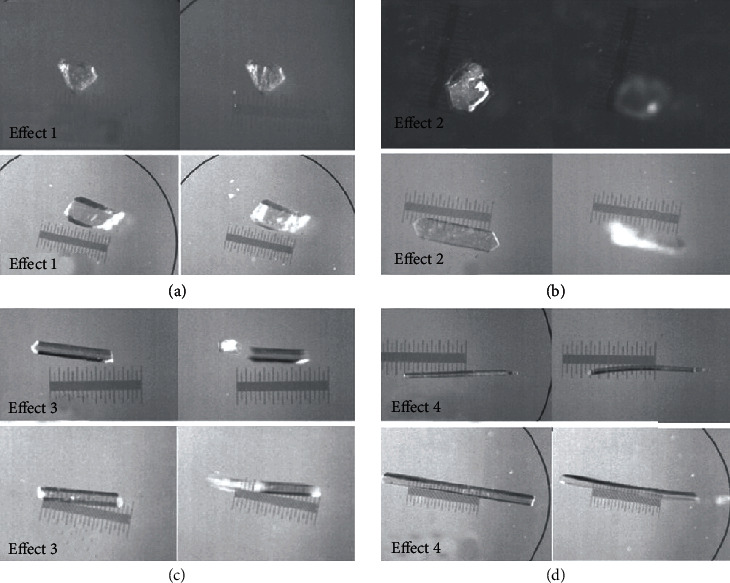
Four photomechanical effects of single crystal 40 (two examples are shown for each effect), (a) cracking, (b) hopping, (c) splitting, and (d) bending (adapted and copyright permission [81] (a–d), American Chemical Society).

In 2015, Nath's group reported a photoluminescence effect (photoinduced transition) of three coordinated complex crystals induced by [2 + 2] photocycloaddition reactions for the first time, like popcorn popping on a hot surface [81]. Unlike other dimerization reactions, these single crystals exploded violently, pushing them to move by several millimeters under weak UV light. The results showed that the accumulated strain energy during dimerization can trigger the rapid structural transformation, which eventually led to the multistep mechanism of crystal disintegration. They also found that one crystal may have multiple mechanical responses. A rare crystal of benzylidefuranone (40) was studied by the analysis technology of bulk sensitivity and surface sensitivity, which showed various types of photodeformable responses, including surface striation and delamination, photo-salient effect (ballistic disintegration and motion), and photoinduced bending by dimerization. It was demonstrated that the type of photodeformable response mainly depended on the aspect ratio of crystals, which can be divided into four kinds of motions (splitting, cracking, hopping, and bending) (Figure 9). In addition, the surface energy gradually increased with the photodimerization. As a result, the surface layer contracted along the preferred direction related to the decrease of molecular distance during the dimerization process, which led to the appearance of striations on the surface. For elongated crystals, light penetrated from a depth on one side of the crystal, causing a macroscopic shrinkage as visible bending; while for massive crystals, radiation led to split and explode.

**Figure 10:**
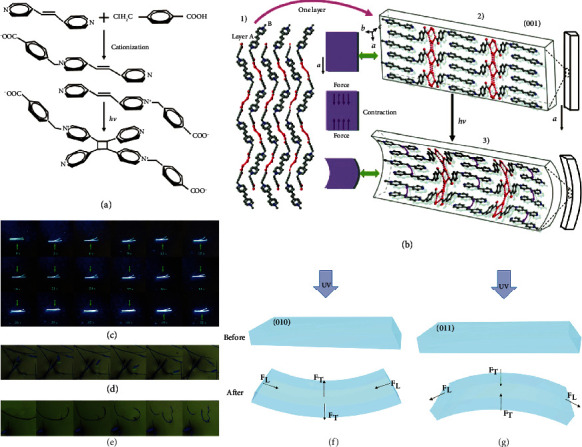
(a) The cationization of one end of the 4,4′-bpe molecule and photocycloaddition. (b) Illustrations of the bending direction of crystal 41 and related crystal structure (adapted and copyright permission [82] (a, b), Wiley-VCH Verlag GmbH & Co. KGaA). (c) Images of the needle-like crystals of 42 before and after UV irradiation for different times (the arrows represent the irradiation direction). (d) Rolling of a small slice and (e) curling of nanofibers of microscopy photographs of crystal 42 before and after UV irradiation (adapted and copyright permission [83] (c-e), Wiley-VCH Verlag GmbH & Co. KGaA). The illustration of the bending of (f) 45 and (g) 47 under UV irradiation.

*Trans*-1,2-bis(4-pyridyl)ethylene(4,4′-bpe) is a kind of photoactive molecule that can undergo the photoinduced [2 + 2] cycloaddition reaction under certain conditions. The photodeformable motion based on the 4,4′-bpe derivative, 1-(4-carboxybenzyl)-4-[2-(4-pyridyl)-vinyl]-pyridinium chloride (41) prepared by substituting the carboxybenzyl group onto one end of the 4,4′-bpe molecule, was reported by Sun and coworkers (Figure 10(a)) [82]. The incorporation of the carboxybenzyl group brought some advantages for the cycloaddition and motion amplification. This needle-like single crystal can be significantly deformed on the centimeter scale upon photoirradiation. The observed photodeformable effect was proposed relating to the [2 + 2] cycloaddition reaction in bpe molecules. Figure 10(b) shows the mechanism of molecular crystal deformation. The photocycloaddition reaction produced a contraction force, leading to an anisotropic contraction on the (001) plane by accumulating and amplifying. The difference of contraction induced crystal bending under the light irradiation, which was similar to the bilayer cantilever effect. It was demonstrated that the deformation scale of single crystal 41 was the largest one among the reported photomechanical materials at that time. Moreover, the deformation process has a high contrast of fluorescence vision, which is an ideal remote detection method for optical mechanical work.

In 2017, Wang and coworkers reported a molecular crystal of (E)-2-(2,4-dichlorostyryl)-benzo[d]oxazole (42), which exhibited obviously interesting photomechanical behaviors [83]. Under UV irradiation, the thinner crystal showed an obvious bending up, while the thicker one bent up slightly. Interestingly, both curved acicular crystals can be straightened with the irradiation from the opposite direction (Figure 10(c)). Moreover, if the crystal was immersed in methanol organogel, the nanofiber would rapidly curl or even form a circle, which then can be rolled into a small block in organic gel under UV light (Figures 10(d) and 10(e)). These photoinduced behaviors were caused by the release of strain in the crystal accumulated by the movement of the atoms during photodimerization. In addition, the styrylbenzoxazole derivatives 43 and 44 were also investigated for comparison, but no photodeformable effect was observed due to the absence of [2 + 2] cycloaddition, confirming the importance of accumulated strain in crystals.

Recently, the same group explored the unique bending behaviors of *trans*-2-(4-fluorostyryl)benzo[d]oxazole (45) and *trans*-2-(2,4-difluorostyryl)benzo[d]oxazole (46) [84]. Both molecules in solid state exhibited obvious aggregation-caused quenching characteristics and unexpected aggregation-induced emission (AIE) through [2 + 2] cycloaddition. It is worth mentioning that the photoisomerization can produce blue and yellow crystals after UV irradiation due to the different packing modes [85]. Moreover, crystals 45 and 47 also have similar photodeformable response. Upon 365 nm irradiation, crystals 45 exhibited an apparent photoinduced bending behavior. It was proposed that during the [2 + 2] cycloaddition, the movements of atoms continuously generated strain in crystals, which was accumulated and only released via mechanical motion. In addition, the photomechanical effect of two more single crystals, (E)-2-(4-fluorostyryl)benzo[d]thiazole (46) and (E)-2-(2,4-difluorostyryl)benzo[d]thiazole (48), was further investigated. Compared with the behaviors of bending away from light in 47 and 48, 45 and 46 crystals showed completely different photoinduced bending behaviors, which bent towards light under UV irradiation. The proton nuclear magnetic resonance spectra before and after light showed that all four crystals 45, 47, 46, and 48 underwent a [2 + 2] cycloaddition reaction in solid state, which was the driven force of converting light energy into mechanical energy. Figures 10(f) and 10(g) illustrate the mechanism of different bending behaviors of 45 and 47, respectively [84]. It showed that molecule 45 was arranged in a “head-to-tail” manner on the photoreactive surface of the crystals. Therefore, when the cycloaddition occurred, the photoreactive surface expanded in the F_T_ direction and shrank in the F_L_ direction, leading to a bending towards the light. In contrast, crystals 47, vertically in a “head-to-head” manner on the photoreaction surface, showed a totally different deformation. The photoreaction surface expanded in the F_L_ direction and shrank in the F_T_ direction forming an upward contraction, which resulted in its bend away behavior. This was mainly due to the crucial impact of C-H···F interaction in their different molecular packing, leading to the positive/negative phototropism of the actuators. This research opens a new door for crystal engineering.

## 6. Triarylethylene-Based Photodeformable Materials

**Scheme 5:**
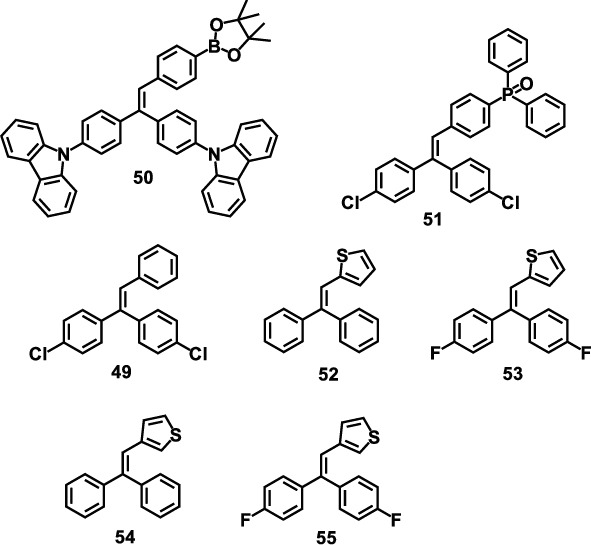
Molecular structures of triarylethylenes discussed in this section.

Diarylethylene-based photochromic materials have made certain progress in the fields of biological detection, photoswitchable molecular devices, optical information storage, and light-controlled self-assembly. However, there are still some challenges remaining, such as complex structures and complicated synthesis steps, which greatly hinder the development of photochromic materials. To simplify molecular structures while maintaining their excellent photochromic properties, the idea of designing and synthesizing triarylethylene photochromic materials was proposed, which can effectively improve poor photochromic performance caused by light isomerization. For triarylethylene, there is no need to embed the double bond in five-membered or six-membered rings when designing molecules, which makes triarylethylene easy to synthesize and modify. Particularly, unlike the systems previously discussed that exhibit evident macroscale photoinduced deformation, some triarylethylene derivatives show photoinduced microinterface properties, whose surface morphology would change under the microscope upon UV irradiation. The chemical structures of triarylethylenes discussed in this section are shown in Scheme 5.

**Figure 11:**
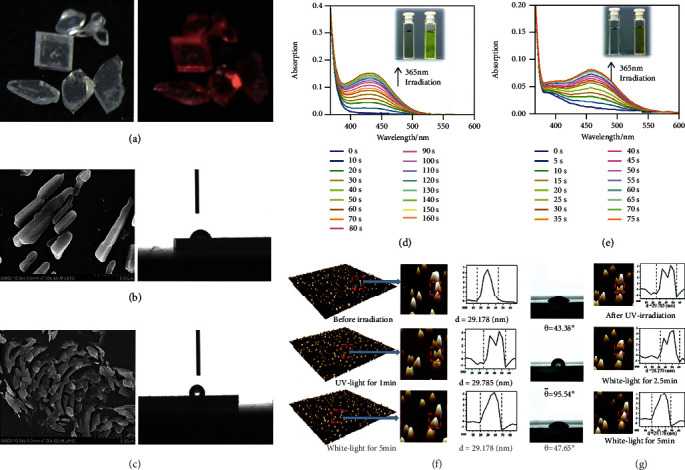
(a) Photographs of crystals 49 before and after irradiation. SEM images of the crystalline surfaces and their contact angles of the water droplets (b) before and (c) after irradiation (adapted and copyright permission [31] (a–c), The Royal Society of Chemistry). Time-dependent UV-Vis absorption spectra of (d) 53 and (e) 55 in degassed THF solution (1.0 × 10^−3^ M) and photographs of their solution before (left) and after (right) UV irradiation. (f) AFM images and contact angles of surface-53 before and after UV irradiation. (g) The morphology reverse process for surface-53 after white light irradiation for different times (adapted and copyright permission [88] (d–g), The Royal Society of Chemistry).

The first triarylethylene-based photochromic material, triphenylethylene derivative (49), was reported by Ou et al. in 2016 [31]. It exhibited excellent photochromic properties, which could change from colorless to red when exposed to 365 nm UV light, while its fluorescence intensity gradually decreased during the photochromic process. After the stoppage of UV irradiation, the emission color changed back to colorless, accompanied by the intensity recovery of fluorescence, as shown in Figure 11(a). In addition, the morphology of crystallite surface of 49 can also be controlled by irradiation (Figures 11(b) and 11(c)). Before irradiation, the crystallites were rod-shaped, and the contact angle of the water droplets on the surface was 73°. After irradiation, 49 grew into scaly crystals, and the contact angle changed. When it changed from 73° to 118°, the wettability of the surface dropped sharply. It was demonstrated that the changes of microinterface in 49 can be ascribed to the formation of the new nuclei, closed-ring isomer, at the beginning of crystal growth.

Subsequently, a carbazole containing triphenylethylene derivative (50) was designed and synthesized in 2017, which exhibited multiple properties including AIE, piezochromism, and photochromism [86]. The piezochromism was related to the twisted packing mode in solid state induced by rigid and bulky groups. The photochromic properties depended on its aggregation state, which can be obviously observed in crystalline state while invisible in amorphous state. This was confirmed by the UV-Vis reflectance spectroscopy and XRD studies. However, the photochromic process of both 49 and 50 only occurred in solid state. To realize the photochromism of triarylethylene both in solution and solid states, Wang et al. further designed and synthesized another molecule 51 by introducing the phosphine-containing heterocycle into the triarylethylene in 2018 [87]. As expected, excellent photochromic properties were observed in 51 both in solution and solid states.

Through the early exploration, it can be found that the photocyclization products of triarylethylene derivatives are instable, which can return to its initial state very quickly. A new molecular design strategy was proposed by replacing phenyl with thienyl unit to improve molecular stability in 2018 [88]. In this work, a series of thienyl-containing triarylethylene derivatives (52, 53, 54, and 55) were synthesized. 53 and 55 exhibited prominent photochromic properties as shown in Figures 11(d) and 11(e), which were found stable in their open-ring structures in darkness for 5 days. Besides, these derivatives can be used to fabricate the real-time and repeatable photoresponsive surface. The nanoaggregates of 53 on SiO_2_ substrate switched from cone-shape to hump-shape under UV irradiation, accompanied by the change of the contact angle of the water droplets on the surface from 43° to 95°. After 5 minutes of white light irradiation, this hump-shaped nanoaggregates 53 gradually became conical, and the contact angle decreased to its initial value (Figure 11(f)). During the reverse process, the real-time and reversible photoresponsive surfaces were observed in surface-53. By controlling white light irradiation time, the intermediate states between ON and OFF can also be achieved, as depicted in Figure 11(g).

By analyzing single crystal structures, it is likely that the distinct morphology changes of triarylethylene derivatives are related to their conformations and molecular stacking modes during photochromic processes. These results are consistent with the photomechanical response of the systems previously discussed and provide an innovative direction for designing new photodeformable molecules in the future.

## 7. Other System-Based Photodeformable Materials

**Scheme 6:**
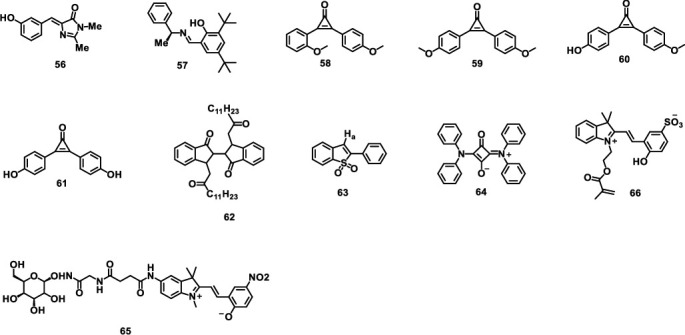
Molecular structures discussed in this section.

Apart from the discussed typical derivatives, many other photodeformable systems based on relatively less studied functional groups were also growing vigorously. The chemical structures of molecules discussed in this section are shown in Scheme 6.

In 2010, Naumov et al. explored the influence of environment on the photophysical and photochemical properties of a green fluorescent protein (GFP) [89], by contacting a large number of chromophores with similar electronic structures but different crystal arrangements. It was demonstrated that the existence of double bonds in another neighborhood can facilitate the cycloaddition reaction. When this approach was accompanied by a wide range of hydrogen bond networks, the bond shrinkage caused by dimerization would lead to an obvious photodeformable effect. Whereas without the proximity, the dimerization would be extinguished, and only isomerization process would occur. The metahydroxy chromophore (56) is the only chromophore analogue of GFP obtained in pure state as multiple stable polymorphs. When exposed to the unfocused weak UV light, crystal 56 would bend, due to the asymmetric substitution of hydrogen bond in donor and acceptor. It was demonstrated that molecular structures determined the accumulation of crystals and thus the reactivity of solids.

Besides, the influence of chirality on the photodeformation was explored by Koshima and Asahi in 2016, by designing platelike crystals of S- and R-enantiomers of photochromic N-3,5-di-tert-butylsalicylidene-1-phenylethylamine (57). Upon the UV irradiation, chiral crystal 57 twisted due to the longitudinal and transverse contraction of the irradiated surface in enol form [90]. According to the crystal structures determined from density functional theory calculations, the molecules of 57 were arranged in a helical manner along the *a* axis on the (00-1) face. When this (00-1) face was irradiated by UV light, the front crystal surface shrank diagonally, while the back crystal surface did not shrink due to a lack of photoisomerization. This led to a platelike crystal twisting toward the incident light in a right-handed helix, as shown in Figure 11(a). In contrast, when the (001) face was under UV irradiation, crystal 57 exhibited a left-handed helix twisting (Figure 11(b)). In addition, the chiroptical and optical anisotropic measurements in 57 indicated that the introduction of chirality diversified the mechanical response of photodeformable crystals.

Later on, four diphenylcyclopropenone crystals (58, 59, 60, and 61) were reported by Gong et al. in 2017, which involved various molecular interactions encoded in individual molecular structures [91]. By analyzing crystal structures and the photoresponsive characteristics of the resultant single-crystal microstrips of these four crystals, it was demonstrated that the magnitude of the intermolecular interaction can effectively regulate the quantum chain reaction and the light-responsive characteristics. Microstrips 58 and 59 with a weak molecular interaction showed an effective chain reaction and a large mechanically optical response, such as optical deformation. In contrast, microstrips 60 and 61 with a strong molecular interaction showed no chain reaction or optical-mechanical effect. The main reason was that the weak molecular interaction created a relatively low energy barrier for the change of molecular volume in the light interaction, which is conducive to the propagation of chain reaction and the generation of large mechanically photoresponsive characteristics. This work provides an innovative way to design molecular crystals with enhanced mechanical photoresponse.

**Figure 12:**
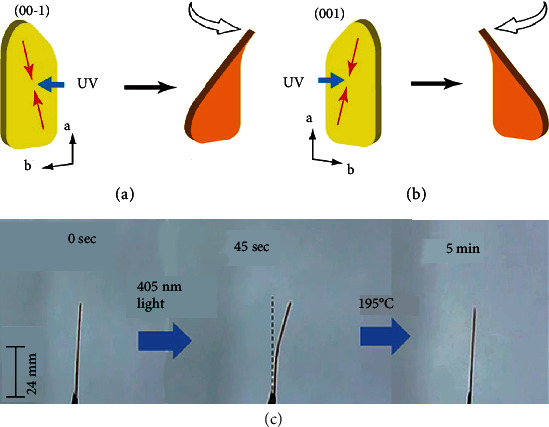
Relationship between the direction of twisting and the face of 63 irradiated with UV light: (a) twist in right-handed helix; (b) twist in left-handed helix (adapted and copyright permission [90] (a, b), Wiley-VCH Verlag GmbH & Co. KGaA). (c) The reversible bending of crystal 62 (adapted and copyright permission [92], American Chemical Society).

Unlike the generally discussed crystals, some interesting phenomena were also reported. In 2018, Samanta and coworkers successfully designed a novel diindene derivative compound (62), which can undergo photopolymerization under visible light and depolymerization after heating [92]. As shown in Figure 12(c), needle crystal 62 showed a photoinduced bending under 405 nm irradiation for 45 s and then recovered by heating at 195°C for 5 min. It revealed that the photodeformation might also result from visible-light-driven polymerization and thermal depolymerization. In addition, not only mechanical behaviors, light irradiation may have concomitant impacts on other properties. For example, a new photoresponsive crystal 63 was reported by Guo et al. in 2019 [93]. Upon UV irradiation, it exhibited not only photodeformation, such as striking, splitting, hopping, and bending, but a significant fluorescence enhancement due to photodimerization. Generally, the formation of cyclobutane ring can result in unfavorable luminescence due to the broken *π*-conjugation. However, aggregation-induced emission was observed in the photodimer of 63 with enhanced fluorescence peaking at 415 nm. It was mainly attributed to the efficient intramolecular through-space conjugation and restriction of intramolecular vibrations. The unique property endowed this kind of photodeformable molecules with promising applications in smart optoelectronic devices and bionic science.

The photodeformable behaviors of molecular crystals can be manipulated by many factors, including the light wavelength, time, density, and irradiation sites. In 2020, De and coworkers designed a new crystal 1,3-bis(diphenylamino)squaraine (64) [47], which exhibited a variety of photoinduced motions, including translating, rotating, and jumping, by controlling the irradiation parameters. Theoretical calculations indicated that these photomechanical responses arose from the release of molecular strain, accumulated by molecular conformation change between the ground and excited state. The well-controlled multiple photoinduced motions brought them great potential in the application of remotely photocontrolled microrobots.

Furthermore, some spiropyran derivatives were reported showing both photochromic and photodeformable properties when combined with gels or liquid crystals, which endowed them with huge application prospects in biomedicine. In 2016, Xiao and coworkers prepared a supramolecular hydrogel consisting of spiropyran (65)-conjugated galactose for photocontrolled delivery of microRNAs. The hydrogel showed great advantage in delivering the released microRNAs to specific cells [94]. Moreover, a new water-soluble spiropyran derivative (66) containing sulfonate groups was designed and synthesized by Li et al. in 2020 [95]. By incorporating into cross-linked polymer networks, the spiropyran hydrogels exhibited a volumetric expansion upon light irradiation, which can recover in the dark. By regulating pH values and polymeric structures, a highly controllable volumetric expansion can be achieved. The light-driven expansion observed in spiropyran hydrogels provides a promising molecular design strategy for mimicking biological functions, such as plant phototaxis and the energy conversion of animal muscles.

## 8. Potential Applications of Photodeformable Crystals

Photodeformable materials have captured enormous attention in both academia and industry owing to their excellent photoinduced properties. The controllable and reversible photodeformation, such as bending, curling, and twisting, can mimic the movement of creatures by utilizing the energy generated from the photoinduced motions, which have been widely used for bionic devices (e.g., artificial muscles and artificial robots). Besides, some photodeformable systems combining photochromic derivatives with liquid crystals or gels also show great potential in biomedical fields for targeted drug delivery.

**Figure 13:**
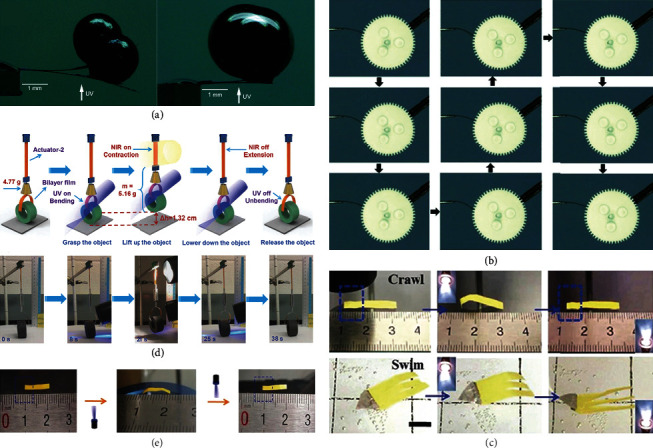
(a) The photodeformable work of cocrystal 4o·FN (adapted and copyright permission [52], American Chemical Society). (b) The rotation of gearwheel hit by the rodlike mixed crystal containing 5 and 6 (adapted and copyright permission [53], Wiley-VCH Verlag GmbH & Co. KGaA). (c) The photographs of crawling and swimming behaviors under UV irradiation (adapted and copyright permission [44], Wiley-VCH Verlag GmbH & Co. KGaA). (d) The photographs and schematic of crane grasping, lifting up, lowering down, and releasing an object (adapted and copyright permission [45], Wiley-VCH Verlag GmbH & Co. KGaA). (e) The photographs of photoactuators fabricated by large single crystals creeping and crawling on the ground (adapted and copyright permission [97], Wiley-VCH Verlag GmbH & Co. KGaA).

In 2010, Morimoto and Irie reported a molecular crystal cantilever made of a cocrystal 4o·FN, which showed a reversible bending motion under alternative UV and visible light [52]. As shown in Figure 13(a), the cocrystal cantilever was able to hold metal balls hundreds of times heavier than itself. The generated maximum stress was over two magnitude orders larger than that of muscles and comparable to that of piezoelectric crystals. Later on, a rodlike mixed crystal containing crystals 5 and 6 was reported by Terao et al., which could bend upon UV irradiation and then hit the gearwheels making them rotate [53] (Figure 13(b)). These crystals can generate strong forces and achieve large mechanical work attributing to a high Young's modulus. Despite the great properties, the molecular rigidity restricted its application for artificial muscles.

By incorporating photodeformable molecular crystals (e.g., anthracenes) to connective polymer (e.g., polyvinylidene fluoride), a simple and generic strategy to prepare artificial muscles named as “mixed-matrix membrane” was proposed by Yu et al. in 2018 [44]. The designed hybrid material exhibits both high Young's modulus and rapid response of molecular crystals, and high elasticity of polymers. The successfully obtained artificial muscles can mimic several muscle movements, like grasping, crawling, and swimming (Figure 13(c)). Instead of polymers, Guo et al. firstly introduced crystalline covalent organic frameworks (COFs) into the photoactuator area. They successfully developed a new strategy to directly install photoresponsive crystals (i.e., acylhydrazone) into the COF skeleton and fabricated a series of uniform membranes with outstanding mechanical properties in 2020 [96]. The designed material not only exhibited excellent flexibility and strength but fast response to UV light. This strategy broadens the development of photoactuators and provides new opportunities constructing the multistimulus responsive materials.

Apart from artificial muscles, soft robot is another typical application of composite materials based on photodeformable crystals. The structural diversity and controllability of photodeformable crystals make them promising for flexible robotic applications. In 2018, Lu and coworkers designed a light-controlled polymer “crane,” composed of a photodeformable nanocomposite, which was synthesized by doping gold nanorods into azobenzene liquid-crystalline dynamic networks [45]. It exhibited robot-like behaviors such as walking, grasping, lifting up, and lowering down upon NIR and UV irradiation (Figure 13(d)). In 2019, Zhang et al. reported an outstanding photoactuator fabricated by large single crystals of platinum-based linear polymer by [4 + 4] cycloaddition of anthracene in a single-crystal-to-single-crystal manner [97]. Attributing to reversible contraction effect of unit-cell volume upon light irradiation, the photoactuator showed robot-like creeping and crawling behaviors on the ground as shown in Figure 13(e).

Except for the UV light, the visible- and NIR-driven photodeformable crystals for the manipulation of micro- or nano-sized objects show great utility in biomedical field. Liu and coworkers reported a NIR light-absorbing molecular crystal by combining an upconverting nanoparticle with integrated azobenzene (29)-modified mesoporous silica [69]. The photoinduced motions of the hybrid molecules facilitated the release of anticancer drugs as well as their targeted delivery, which can also be controlled by the NIR irradiation intensity. Photodeformable crystals can also be used as biomaterials that can interact with cells in biomedical field. In 2018, Pennacchio et al. developed an acrylamide-modified gelatin hydrogel based on an azobenzene-based crosslinker. It exhibited a sensitively light-triggered expansion, which led to an in-plane nuclear deformation of physically confined immortalized fibroblasts [46]. This work promotes the development of photoresponsive gelatin for cell culture applications.

## 9. Summary and Outlook

In summary, the current progress of typical photodeformable organic crystals, including diarylethenes, azobenzenes, anthracenes, olefins, triarylethylenes, and their derivatives, as well as their photoreaction mechanisms and promising applications is reviewed. Molecular crystals with different structures tend to have different photodeformable behaviors, such as bending, twisting, hopping, curling, and bursting. Their corresponding photomechanical behaviors, excitation conditions, and color changes are summarized in Table 1. In diarylethenes, a photoisomerization process generally occurs with crystals changing from open-ring to closed-ring isomers upon UV irradiation. The diverse magnitudes of stacking changes in the crystal surfaces and inside the crystal result in the photoinduced deformation. Different from diarylethenes, azobenzene derivatives are a kind of photomechanical molecules that have a photodeformable mechanism of trans-cis isomerization. They exhibit stable photostability and fast response under light irradiation. For anthracenes and olefins, photodeformable effect mainly derives from the solid-state photocycloaddition reactions, including [2 + 2] and [4 + 4] reactions, which can induce significant change of molecular size and spatial structures. In addition, unlike previously discussed systems with macroscopic deformation, triarylethylenes exhibit photoinduced morphological changes on a microscopic level because of the drastic conformational changes and molecular stacking mode changes during photochromic processes. Therefore, the challenge of obtaining a photodeformable crystal lies not only in the stacking arrangements of photosensitive chromophores but also in the packing distance of the chromophores. In addition to the aforementioned crystal systems, there are more photodeformable systems worth exploring.

With the joint efforts of numerous researchers, the study of photoderformable crystals has achieved impressive progresses, particularly in the development of materials based on the photocyclizations (e.g., diarylethenes and triarylethylenes), photoisomers (e.g., azobenzenes), and photodimerizations (e.g., anthracenes and olefins). Some of them also exhibit multiple stimulus responses, such as heat, pH, and forces. The excellent mechanical response of molecules to light irradiation provides a great potential in the applications of photoactuators, molecular rotors, molecular robots, artificial muscles, biomedicine, photoswitches, micro- and nanomechanical devices, etc. However, pure molecular crystals are still faced with problems of poor stability, controllability, and inflexibility, which can hardly meet the needs of practical applications. Therefore, there is still plenty of space for valuable research directions to improve the applicability. First of all, it is crucial to design and synthesize photodeformable crystals towards excellent mechanical properties, such as high efficiency, fast response, high fatigue resistance, and flexibility, which are essential for the applications of photoactuators and artificial intelligent muscles. Secondly, how to improve the controllability of mechanical response of photodeformable crystals is also of great challenge. Only by effectively controlling the responsive type, direction, and degree of mechanical motions could photodeformable crystals be widely taken advantage of. Thirdly, as the intrinsic photodeformation occurs by converting photon energy into molecular macro- or micromechanical energy, how to improve the energy conversion rate is extremely significant for the applications. Fourthly, nanoscale photodeformable microcrystals should be taken into account seriously by researchers due to their performances in micro- and nanomechanical devices. Moreover, achieving excitable photodeformation upon visible light or NIR light irradiation is also a potential research direction of photodeformable crystals, as there are few relevant studies at present, which will greatly diversify their applications. Last but not the least, pure molecular crystals are found hard to be directly used in many complex practical applications due to their inherent physical properties, such as low water solubility, poor biocompatibility, and biodegradability. To develop more high-performance and diverse applications, the exploration of photodeformable materials can be expanded from small molecular crystals to polymeric materials, such as polymers and COFs. Combing all the advantages of photodeformable materials, designing hybrid and composite materials by introducing photodeformable groups into gels, liquid crystals, polymers, or COFs is also of great prospect to develop promising candidates for the applications in bionic and biomedical fields, such as photoactuators, soft robots, and anticancer carriers. To address all these issues, additional efforts need to be made to comprehensively understand the fundamental scientific mechanisms and promote the material system explorations. We hope this review could provide a basic guidance for further development of photomechanical materials.

## Figures and Tables

**Table 1:** The summary of photodeformable molecular systems and corresponding properties.

Systems	Molecular crystals	*λ*_excitation_/*λ*_recovery_ (nm)	Photodeformable behaviors	Color changes
Diarylethenes	1	*λ* =365 / *λ* >500	Bend	Colorless ↔ blue
	2	*λ* = 365/*λ* > 500	Bend	Colorless ↔ purple
	3	*λ* = 365/*λ* > 480	Bend	Colorless ↔ blue
	4·FN^a^	*λ* = 365/*λ* > 440	Bend	Colorless ↔ blue
	5 : 6^b^	*λ* = 365/*λ* > 500	Bend	n^c^
	7	*λ* = 254/*λ* > 500	Bend, roll	n
	8	*λ* = 254/*λ* > 500	Bend	n
	9	*λ* = 365/*λ* > 500	Twist	Colorless ↔ blue
	10	*λ* = 365/*λ* > 500	Twist	Colorless ↔ blue
	11	*λ* = 365/*λ* > 500	Burst	Colorless ↔ blue
	12	*λ* = 365/*λ* > 500	Burst	Colorless ↔ blue
	13	*λ* = 313/*λ* > 500	Bend	Colorless ↔ blue
	14	*λ* = 313/*λ* = 580	Bend	Colorless ↔ red
	15	*λ* = 394/*λ* = 532	Bend	Colorless ↔ blue

Azobenzenes	16	*λ* = 365/n	Bend	n
	17	*λ* = 360/*λ* = 445	Bend	n
	18	*λ* < 400/n	Twist, bend	n
	19	*λ* = 457/n	Bend	n
	20	*λ* > 400/n	Bend	n
	21	*λ* > 400/n	Bend	n
	22	*λ* > 400/n	Bend	n
	23	*λ* > 400/n	Bend	n
	24	*λ* > 400/n	Bend	n
	25	*λ* = 457/n	Bend	Yellow ↔ orange-red
	26	*λ* = 457/n	Bend	n
	27	*λ* < 400/n	Bend	n
	28	*λ* = 980/*λ* = 470	Bend	n
	29	*λ* = 980/n	Rotation	n
	30	*λ* > 400/n	n	n

Anthracenes	31	*λ* = 365/n	Bend	n
	32	*λ* > 300/n	Bend, twist	n
	33	n	n	n
	34	n	Bend, twist	n
	35	*λ* = 405/n	Bend, twist	n
	36	*λ* = 440/n	Bend, twist	n
	37	*λ* = 365/n	Bend, twist	n
	38	*λ* > 450/n	Curl	n
	39	*λ* > 530/*λ* < 405	Bend	n

Olefins	40	*λ* = 365/n	Bend, crack, split, hop	n
	41	*λ* = 365/n	Bend	n
	42	*λ* = 365/n	Bend, curl, roll, salient	n
	43	n	n	n
	44	n	n	n
	45	*λ* = 365/n	Bend	n
	46	*λ* = 365/n	Bend	n
	47	*λ* = 365/n	Bend	n
	48	*λ* = 365/n	Bend	n

Triarylethylenes	49	*λ* = 365/n	Micromorphological changes	Colorless ↔ red
	50	*λ* = 365/n	n	Colorless ↔ red
	51	*λ* = 365/n	n	Colorless ↔ yellow
	52	n	n	n
	53	*λ* = 365/n	Micromorphological changes	Colorless ↔ yellow
	54	n	n	n
	55	*λ* = 365/n	Micromorphological changes	Colorless ↔ yellow

Others	56	*λ* = 365/n	Bend, hop	n
	57	*λ* < 400/n	Twist	n
	58	*λ* = 375/n	Melt	n
	59	*λ* = 375/n	Bend	n
	60	n	n	n
	61	n	n	n
	62	*λ* = 405/n	Bend	n
	63	*λ* = 365/n	Strike, split, hop, bend	n
	64	*λ* = 400/n	Move, rotate, Flip	n
	65	*λ* > 400/n	n	Red ↔ yellow
	66	*λ* = 425/n	Bend	n

^a^4·FN represents cocrystal of 4 and perfluoronaphthalene (FN); ^b^5 : 6 represents two-component mixed crystal containing 5 and 6 (5 : 6 = 63/37); ^c^“n” represents “not mentioned in the paper or none of the property”.

## References

[1] Yagai S., Okamura S., Nakano Y. (2014). Design amphiphilic dipolar *π*-systems for stimuli-responsive luminescent materials using metastable states. *Nature Communications*.

[2] Yan D., Yang H., Meng Q., Lin H., Wei M. (2014). Two-component molecular materials of 2,5-diphenyloxazole exhibiting tunable ultraviolet/blue polarized emission, pump-enhanced luminescence, and mechanochromic response. *Advanced Functional Materials*.

[3] Yang J., Fang M., Li Z. (2020). Stimulus-responsive room temperature phosphorescence in purely organic luminogens. *InfoMat*.

[4] Irie M., Fukaminato T., Matsuda K., Kobatake S. (2014). Photochromism of diarylethene molecules and crystals: memories, switches, and actuators. *Chemical Reviews*.

[5] Xie Y., Li Z. (2018). Triboluminescence: recalling interest and new aspects. *Chem*.

[6] Jia S., Fong W.-K., Graham B., Boyd B. J. (2018). Photoswitchable molecules in long-wavelength light-responsive drug delivery: from molecular design to applications. *Chemistry of Materials*.

[7] Thévenot J., Oliveira H., Sandre O., Lecommandoux S. (2013). Magnetic responsive polymer composite materials. *Chemical Society Reviews*.

[8] Jiang K., Wang Y., Cai C., Lin H. (2018). Conversion of carbon dots from fluorescence to ultralong room-temperature phosphorescence by heating for security applications. *Advanced Materials*.

[9] Wang D., Imae T. (2004). Fluorescence emission from dendrimers and its ph dependence. *Journal of the American Chemical Society*.

[10] Naumov P., Chizhik S., Panda M. K., Nath N. K., Boldyreva E. (2015). Mechanically responsive molecular crystals. *Chemical Reviews*.

[11] Zhao H., Sterner E. S., Coughlin E. B., Theato P. (2012). *O*-Nitrobenzyl alcohol derivatives: opportunities in polymer and materials science. *Macromolecules*.

[12] Chatani S., Kloxin C. J., Bowman C. N. (2014). The power of light in polymer science: photochemical processes to manipulate polymer formation, structure, and properties. *Polymer Chemistry*.

[13] Wales D. J., Cao Q., Kastner K., Karjalainen E., Newton G. N., Sans V. (2018). 3D-printable photochromic molecular materials for reversible information storage. *Advanced Materials*.

[14] Guo X., Zhang D., Zhu D. (2004). Logic control of the fluorescence of a new dyad, spiropyran-perylene Diimide-Spiropyran, with light, ferric ion, and proton: construction of a new Three-Input“AND” logic gate. *Advanced Materials*.

[15] Raymo F. M., Alvarado R. J., Giordani S., Cejas M. A. (2003). Memory effects based on intermolecular photoinduced proton transfer. *Journal of the American Chemical Society*.

[16] Li H., Pu S., Liu G., Liu W., Yao B. (2010). Polarization holographic optical recording based on a new photochromic diarylethene compound. *Frontiers of Chemistry in China*.

[17] Qi Q., Li C., Liu X. (2017). Solid-state photoinduced luminescence switch for advanced anticounterfeiting and super-resolution imaging applications. *Journal of the American Chemical Society*.

[18] Huang G., Xia Q., Huang W. (2019). Multiple anti-counterfeiting guarantees from a simple tetraphenylethylene derivative – high-contrasted and multi-state mechanochromism and photochromism. *Angewandte Chemie International Edition*.

[19] Li Y., Duan Y., Li J., Zheng J., Yu H., Yang R. (2012). Simultaneous nucleophilic-substituted and electrostatic interactions for thermal switching of spiropyran: a new approach for rapid and selective colorimetric detection of thiol-containing amino acids. *Analytical Chemistry*.

[20] Champagne B., Plaquet A., Pozzo J.-L., Rodriguez V., Castet F. (2012). Nonlinear optical molecular switches as selective cation sensors. *Journal of the American Chemical Society*.

[21] Zhu L., Wu W., Zhu M.-Q., Han J. J., Hurst J. K., Li A. D. Q. (2007). Reversibly photoswitchable dual-color fluorescent nanoparticles as new tools for live-cell imaging. *Journal of the American Chemical Society*.

[22] Shao N., Jin J., Wang H. (2010). Design of bis-spiropyran ligands as dipolar molecule receptors and application to in vivo glutathione fluorescent probes. *Journal of the American Chemical Society*.

[23] Armistead W. H., Stookey S. D. (1964). Photochromic silicate glasses sensitized by silver halides. *Science*.

[24] Yam V. W.-W., Ko C.-C., Zhu N. (2004). Photochromic and luminescence switching properties of a versatile diarylethene-containing 1,10-phenanthroline ligand and its rhenium(I) complex. *Journal of the American Chemical Society*.

[25] Bandara H. M. D., Burdette S. C. (2012). Photoisomerization in different classes of azobenzene. *Chemical Society Reviews*.

[26] Paramonov S. V., Lokshin V., Fedorova O. A. (2011). Spiropyran, chromene or spirooxazine ligands: insights into mutual relations between complexing and photochromic properties. *Journal of Photochemistry and Photobiology C: Photochemistry Reviews*.

[27] Meng X., Gui B., Yuan D., Zeller M., Wang C. (2016). Mechanized azobenzene-functionalized zirconium metal-organic framework for on-command cargo release. *Science Advances*.

[28] Li C., Xiong K., Chen Y. (2020). Visible-light-driven photoswitching of aggregated-induced emission-active diarylethenes for super-resolution imaging. *ACS Applied Materials & Interfaces*.

[29] Jeong W., Khazi M. I., Park D.-H., Jung Y.-S., Kim J.-M. (2016). Full color light responsive diarylethene inks for reusable paper. *Advanced Functional Materials*.

[30] Wu N. M.-W., Ng M., Lam W. H., Wong H.-L., Yam V. W.-W. (2017). Photochromic heterocycle-fused thieno[3,2-b]phosphole oxides as visible light switches without sacrificing photoswitching efficiency. *Journal of the American Chemical Society*.

[31] Ou D., Yu T., Yang Z. (2016). Combined aggregation induced emission (AIE), photochromism and photoresponsive wettability in simple dichloro-substituted triphenylethylene derivatives. *Chemical Science*.

[32] Wu N. M., Ng M., Yam V. W. (2019). Photochromic benzo[b]phosphole alkynylgold(I) complexes with mechanochromic property to serve as multistimuli-responsive materials. *Angewandte Chemie International Edition*.

[33] Garcia-Garibay M. A. (2007). Molecular crystals on the move: from single-crystal-to-single-crystal photoreactions to molecular machinery. *Angewandte Chemie International Edition*.

[34] Irie M. (2008). Photochromism and molecular mechanical devices. *Bulletin of the Chemical Society of Japan*.

[35] Lange C. W., Foldeaki M., Nevodchikov V. I., Cherkasov K., Abakumov G. A., Pierpont C. G. (1992). Photomechanical properties of rhodium(I)-semiquinone complexes. The structure, spectroscopy, and magnetism of (3,6-di-tert-butyl-1,2-semiquinonato)dicarbonylrhodium(I). *Journal of the American Chemical Society*.

[36] Yu Y., Nakano M., Ikeda T. (2003). Directed bending of a polymer film by light. *Nature*.

[37] Ikeda T., Mamiya J.-I., Yu Y. (2007). Photomechanics of liquid-crystalline elastomers and other polymers. *Angewandte Chemie International Edition*.

[38] Alexa H. K., Kuenstler S., Hayward R. C. (2019). Liquid crystal elastomer waveguide actuators. *Advanced Materials*.

[39] Lansakara T. I., Tong F., Bardeen C. J., Tivanski A. V. (2020). Mechanical properties and photomechanical fatigue of macro- and nanodimensional diarylethene molecular crystals. *Nano Letters*.

[40] Zhao Y., Ippolito S., Samorì P. (2019). Functionalization of 2D materials with photosensitive molecules: from light-responsive hybrid systems to multifunctional devices. *Advanced Optical Materials*.

[41] Wu K., Sun J., Ma Y. (2020). Spatiotemporal regulation of dynamic cell microenvironment signals based on an azobenzene photoswitch. *Journal of Materials Chemistry B*.

[42] Chang V. Y., Fedele C., Priimagi A., Shishido A., Barrett C. J. (2019). Photoreversible soft azo dye materials: toward optical control of bio-interfaces. *Advanced Optical Materials*.

[43] Puliafito A., Ricciardi S., Pirani F. (2019). Driving cells with light-controlled topographies. *Advanced Science*.

[44] Yu Q., Yang X., Chen Y. (2018). Fabrication of light-triggered soft artificial muscles via a mixed-matrix membrane strategy. *Angewandte Chemie International Edition*.

[45] Lu X., Zhang H., Fei G. (2018). Liquid-crystalline dynamic networks doped with gold nanorods showing enhanced photocontrol of actuation. *Advanced Materials*.

[46] Pennacchio F. A., Fedele C., de Martino S., Cavalli S., Vecchione R., Netti P. A. (2018). Three-dimensional microstructured azobenzene-containing gelatin as a photoactuable cell confining system. *ACS Applied Materials & Interfaces*.

[47] de J., Liao Q., Xiao X. (2020). Remotely photocontrolled microrobots based on photomechanical molecular crystals. *ACS Applied Materials & Interfaces*.

[48] Zheng K., Han S., Zeng X. (2018). Rewritable optical memory through high-registry orthogonal upconversion. *Advanced Materials*.

[49] Erbas-Cakmak S., Leigh D. A., McTernan C. T., Nussbaumer A. L. (2015). Artificial molecular machines. *Chemical Reviews*.

[50] Kobatake S., Takami S., Muto H., Ishikawa T., Irie M. (2007). Rapid and reversible shape changes of molecular crystals on photoirradiation. *Nature*.

[51] Kuroki L., Takami S., Yoza K., Morimoto M., Irie M. (2010). Photoinduced shape changes of diarylethene single crystals: correlation between shape changes and molecular packing. *Photochemical & Photobiological Sciences*.

[52] Morimoto M., Irie M. (2010). A diarylethene cocrystal that converts light into mechanical work. *Journal of the American Chemical Society*.

[53] Terao F., Morimoto M., Irie M. (2012). Light-driven molecular-crystal actuators: rapid and reversible bending of rodlike mixed crystals of diarylethene derivatives. *Angewandte Chemie International Edition*.

[54] Kitagawa D., Nishi H., Kobatake S. (2013). Photoinduced twisting of a photochromic diarylethene crystal. *Angewandte Chemie International Edition*.

[55] Kitagawa D., Tsujioka H., Tong F., Dong X., Bardeen C. J., Kobatake S. (2018). Control of photomechanical crystal twisting by illumination direction. *Journal of the American Chemical Society*.

[56] Uchida K., Sukata S., Matsuzawa Y. (2008). Photoresponsive rolling and bending of thin crystals of chiral diarylethenes. *Chemical Communications*.

[57] Kitagawa D., Okuyama T., Tanaka R., Kobatake S. (2016). Photoinduced rapid and explosive fragmentation of diarylethene crystals having urethane bonding. *Chemistry of Materials*.

[58] Kitagawa D., Kawasaki K., Tanaka R., Kobatake S. (2017). Mechanical behavior of molecular crystals induced by combination of photochromic reaction and reversible single-crystal-to-single-crystal phase transition. *Chemistry of Materials*.

[59] Kajiya R., Sakakibara S., Ikawa H. (2019). Inorganic–organic hybrid photomechanical crystals consisting of diarylethenes and cage siloxanes. *Chemistry of Materials*.

[60] Dong X., Guo T., Kitagawa D., Kobatake S., Palffy-Muhoray P., Bardeen C. J. (2019). Effects of template and molecular nanostructure on the performance of organic–inorganic photomechanical actuator membranes. *Advanced Functional Materials*.

[61] Chen M., Yao B., Kappl M. (2019). Entangled azobenzene-containing polymers with photoinduced reversible solid-to-liquid transitions for healable and reprocessable photoactuators. *Advanced Functional Materials*.

[62] Koshima H., Ojima N., Uchimoto H. (2009). Mechanical motion of azobenzene crystals upon photoirradiation. *Journal of the American Chemical Society*.

[63] Cheng S. C., Chen K. J., Suzaki Y. (2018). Reversible laser-induced bending of pseudorotaxane crystals. *Journal of the American Chemical Society*.

[64] Gupta P., Karothu D. P., Ahmed E., Naumov P., Nath N. K. (2018). Thermally twistable, photobendable, elastically deformable, and self-healable soft crystals. *Angewandte Chemie International Edition*.

[65] Bushuyev O. S., Singleton T. A., Barrett C. J. (2013). Fast, reversible, and general photomechanical motion in single crystals of various azo compounds using visible light. *Advanced Materials*.

[66] Bushuyev O. S., Tomberg A., Friscic T., Barrett C. J. (2013). Shaping crystals with light: crystal-to-crystal isomerization and photomechanical effect in fluorinated azobenzenes. *Journal of the American Chemical Society*.

[67] Nath N. K., Pejov L., Nichols S. M. (2014). Model for photoinduced bending of slender molecular crystals. *Journal of the American Chemical Society*.

[68] Wu W., Yao L., Yang T., Yin R., Li F., Yu Y. (2011). NIR-light-induced deformation of cross-linked liquid-crystal polymers using upconversion nanophosphors. *Journal of the American Chemical Society*.

[69] Liu J., Bu W., Pan L., Shi J. (2013). NIR-triggered anticancer drug delivery by upconverting nanoparticles with integrated azobenzene-modified mesoporous silica. *Angewandte Chemie International Edition*.

[70] Paternò G. M., Colombo E., Vurro V. (2020). Membrane environment enables ultrafast isomerization of amphiphilic azobenzene. *Advanced Science*.

[71] Al-Kaysi R. O., Muller A. M., Bardeen C. J. (2006). Photochemically driven shape changes of crystalline organic nanorods. *Journal of the American Chemical Society*.

[72] Chalek K. R., Dong X., Tong F. (2021). Bridging photochemistry and photomechanics with NMR crystallography: the molecular basis for the macroscopic expansion of an anthracene ester nanorod. *Chemical Science*.

[73] Al-Kaysi R. O., Bardeen C. J. (2007). Reversible photoinduced shape changes of crystalline organic nanorods. *Advanced Materials*.

[74] Good J. T., Burdett J. J., Bardeen C. J. (2009). Using two-photon excitation to control bending motions in molecular-crystal nanorods. *Small*.

[75] Zhu L., Tong F., Salinas C. (2014). Improved solid-state photomechanical materials by fluorine substitution of 9-anthracene carboxylic acid. *Chemistry of Materials*.

[76] Zhu L., Al-Kaysi R. O., Bardeen C. J. (2016). Photoinduced ratchet-like rotational motion of branched molecular crystals. *Angewandte Chemie International Edition*.

[77] Kim T., Zhu L., Mueller L. J., Bardeen C. J. (2014). Mechanism of photoinduced bending and twisting in crystalline microneedles and microribbons composed of 9-methylanthracene. *Journal of the American Chemical Society*.

[78] Tong F., Xu W., al-Haidar M., Kitagawa D., al-Kaysi R. O., Bardeen C. J. (2018). Photomechanically induced magnetic field response by controlling molecular orientation in 9-methylanthracene microcrystals. *Angewandte Chemie International Edition*.

[79] Kim T., Al-Muhanna M. K., Al-Suwaidan S. D., Al-Kaysi R. O., Bardeen C. J. (2013). Photoinduced curling of organic molecular crystal nanowires. *Angewandte Chemie International Edition*.

[80] Zhu L., Tong F., Zaghloul N., Baz O., Bardeen C. J., al-Kaysi R. O. (2016). Characterization of a P-type photomechanical molecular crystal based on the E → Z photoisomerization of 9-divinylanthracene malonitrile. *Journal of Materials Chemistry C*.

[81] Nath N. K., Runčevski T., Lai C. Y., Chiesa M., Dinnebier R. E., Naumov P. (2015). Surface and bulk effects in photochemical reactions and photomechanical effects in dynamic molecular crystals. *Journal of the American Chemical Society*.

[82] Sun J. K., Li W., Chen C., Ren C. X., Pan D. M., Zhang J. (2013). Photoinduced bending of a large single crystal of a 1,2-bis(4-pyridyl)ethylene-based pyridinium salt powered by a [2+2] cycloaddition. *Angewandte Chemie International Edition*.

[83] Wang H., Chen P., Wu Z., Zhao J., Sun J., Lu R. (2017). Bending, curling, rolling, and salient behavior of molecular crystals driven by [2+2] cycloaddition of a styrylbenzoxazole derivative. *Angewandte Chemie International Edition*.

[84] Wang H., Liu J., Ye K. (2020). Positive/negative phototropism: controllable molecular actuators with different bending behavior. *CCS Chemistry*.

[85] Wang H., Xing H., Gong J. (2020). “Living” luminogens: light driven ACQ-to-AIE transformation accompanied with solid-state actuation. *Materials Horizons*.

[86] Yu T., Ou D., Wang L. (2017). A new approach to switchable photochromic materials by combining photochromism and piezochromism together in an AIE-active molecule. *Materials Chemistry Frontiers*.

[87] Wang L., Yu T., Xie Z. (2018). Gated photochromic molecules with AIEgen: turn-on the photochromism with an oxidation reagent. *RSC Advances*.

[88] Wang L., Yu T., Xie Z. (2018). Design, synthesis and photochromism studies of thienyl containing triarylethylene derivatives and their applications in real-time photoresponsive surfaces. *Journal of Materials Chemistry C*.

[89] Naumov P., Kowalik J., Solntsev K. M. (2010). Topochemistry and photomechanical effects in crystals of green fluorescent protein-like chromophores: effects of hydrogen bonding and crystal packing. *Journal of the American Chemical Society*.

[90] Takanabe A., Tanaka M., Johmoto K. (2016). Optical activity and optical anisotropy in photomechanical crystals of chiral salicylidenephenylethylamines. *Journal of the American Chemical Society*.

[91] Gong Y., Zhang Y., Xiong W., Zhang K., Che Y., Zhao J. (2017). Molecular interactions control quantum chain reactions toward distinct photoresponsive properties of molecular crystals. *Journal of the American Chemical Society*.

[92] Samanta R., Ghosh S., Devarapalli R., Reddy C. M. (2018). Visible light mediated photopolymerization in single crystals: photomechanical bending and thermomechanical unbending. *Chemistry of Materials*.

[93] Guo J., Fan J., Liu X., Zhao Z., Tang B. Z. (2020). Photomechanical luminescence from through-space conjugated AIEgens. *Angewandte Chemie International Edition*.

[94] Xiao X., Hu J., Wang X. (2016). A dual-functional supramolecular hydrogel based on a spiropyran–galactose conjugate for target-mediated and light-controlled delivery of microRNA into cells. *Chemical Communications*.

[95] Li C., Iscen A., Palmer L. C., Schatz G. C., Stupp S. I. (2020). Light-driven expansion of spiropyran hydrogels. *Journal of the American Chemical Society*.

[96] Guo X., Mao T., Wang Z. (2020). Fabrication of photoresponsive crystalline artificial muscles based on pegylated covalent organic framework membranes. *ACS Central Science*.

[97] Yu Q., Li M., Gao J. (2019). Fabrication of large single crystals for platinum-based linear polymers with controlled-release and photoactuator performance. *Angewandte Chemie International Edition*.

